# Multifunctional Integrated Nanozymes Facilitate Spinal Cord Regeneration by Remodeling the Extrinsic Neural Environment

**DOI:** 10.1002/advs.202205997

**Published:** 2023-01-16

**Authors:** Tiandi Xiong, Keni Yang, Tongtong Zhao, Haitao Zhao, Xu Gao, Zhifeng You, Caixia Fan, Xinyi Kang, Wen Yang, Yan Zhuang, Yanyan Chen, Jianwu Dai

**Affiliations:** ^1^ School of Nano Technology and Nano Bionics University of Science and Technology of China Hefei 230026 China; ^2^ Key Laboratory for Nano‐Bio Interface Research Division of Nanobiomedicine Suzhou Institute of Nano‐Tech and Nano‐Bionics Chinese Academy of Sciences Suzhou 215123 China; ^3^ State Key Laboratory of Molecular Development Biology Institute of Genetics and Developmental Biology Chinese Academy of Sciences Beijing 100101 China

**Keywords:** anti‐inflammation, antioxidation, catalytic cascade reaction, spinal cord injury

## Abstract

High levels of reactive oxygen species (ROS) and inflammation create a complicated extrinsic neural environment that dominates the initial post‐injury period after spinal cord injury (SCI). The compensatory pathways between ROS and inflammation limited the efficacy of modulating the above single treatment regimen after SCI. Here, novel “nanoflower” Mn_3_O_4_ integrated with “pollen” *^IRF‐5^SiRNA* was designed as a combination antioxidant and anti‐inflammatory treatment after SCI. The “nanoflower” and “pollen” structure was encapsulated with a neutrophil membrane for protective and targeted delivery. Furthermore, valence‐engineered nanozyme Mn_3_O_4_ imitated the cascade response of antioxidant enzymes with a higher substrate affinity compared to natural antioxidant enzymes. Nanozymes effectively catalyzed ROS to generate O_2_, which is advantageous for reducing oxidative stress and promoting angiogenesis. The screened “pollen” *^IRF‐5^SiRNA* could reverse the inflammatory phenotype by reducing interferon regulatory factors‐5 (IRF‐5) expression (protein level: 73.08% and mRNA level: 63.10%). The decreased expression of pro‐inflammatory factors reduced the infiltration of inflammatory cells, resulting in less neural scarring. In SCI rats, multifunctional nanozymes enhanced the proliferation of various neuronal subtypes (motor neurons, interneurons, and sensory neurons) and the recovery of locomotor function, demonstrating that the remodeling of the extrinsic neural environment is a promising strategy to facilitate nerve regeneration.

## Graphical abstract

The novel technique of “nanoflower” Mn_3_O_4_ integrated “pollen” *^IRF‐5^SiRNA*, followed by neutrophil membrane encapsulation, results in anti‐oxidative and anti‐inflammatory benefits while avoiding the formation of compensatory pathways between reactive oxygen species and inflammation. The therapy impact of multifunctional nanozymes demonstrates that remodeling the extrinsic neural environment can facilitate the landscape of nerve regeneration after spinal cord injury.

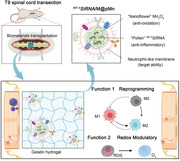

## 1 Introduction

Spinal cord injury (SCI) occurs when an external physical impact (e.g., car accidents, falls, sports injuries, or violent trauma) severely damages the spinal cord, disrupts the neuronal circuitry, and results in variable degrees of irreversible neurological dysfunction.^[^ ^1^ ^]^ Initial mechanical trauma to the spinal cord initiates a secondary injury cascade,^[^ ^2^ ^]^ including ischemia/reperfusion injury, inflammation, reactive oxygen species (ROS) generation, and glutamate‐mediated excitotoxicity,^[^ ^3^ ^]^ which frequently have worse effects than the initial traumatic injury. The harsh extrinsic neural environment at the injury site leads to reduced neuronal survival, resulting in functional connectivity failure.^[^ ^1^, ^3^ ^]^ Hence, an innovative strategy of remodeling the extrinsic neural environment can efficiently boost their regeneration potential.

Blood vessel rupture after SCI could lead to insufficient oxygen supply and further ROS production, which is one of the main inhibitory factors of the extrinsic neural environment in SCI.^[^ ^4^ ^]^ Neurons in the spinal cord are particularly prone to oxidative and electrophilic stress due to their high polyunsaturated fatty acid content and relatively low antioxidant capacity.^[^ ^5^ ^]^ ROS can also cause substantial damage by mediating lipid peroxidation, protein nitration, and the consumption of nitric oxide.^[^ ^6^ ^]^ The relevance of ROS in the pathophysiology of SCI has inspired extensive research on the neuroprotective effects of antioxidants. Nukolova et al. incorporated superoxide dismutase (SOD1) into a polyion complex that could be administered to minimize edema and post‐traumatic cyst formation.^[^ ^7^ ^]^ Ji et al. found that iridium metal complexes scavenge ROS by upregulating SOD1 in injured neurons.^[^ ^8^ ^]^ Nonetheless, the strategy of conjugating SOD with nanocarriers or SOD expression control might fail in the dynamic and complex neuroinhibitory microenvironment, which may be attributed to their limited operational stability, off‐target nature, and monotonous function.^[^ ^9^ ^]^ Therefore, the development of novel antioxidant drug alternatives to alleviate oxidative stress after SCI is urgently required.

Neuroinflammation is another inhibitory component of the extrinsic neural environment in SCI.^[^ ^10^ ^]^ SCI initiates a robust immune response characterized in part by the coordinated infiltration of peripheral leukocytes (particularly M1 macrophages) and their synthesis of cytokines and chemokines. Persistent inflammatory cell infiltration causes further cell death and the formation of cystic microcavities. As the main effectors, macrophages are the target cells for effectively regulating neuroinflammation following SCI.^[^ ^11^ ^]^ Previous studies have demonstrated that the switch from an inflammatory (M1) to a reparative (M2) phenotype is crucial for resolving inflammation.^[^ ^12^ ^]^ Breakthrough research on macrophages has identified several interferon regulatory factors (IRFs) that operate as central switches to activate subsets of M1 or M2 genes, thereby promoting polarization.^[^ ^13^ ^]^ Based on the aforementioned molecular processes, it is a promising avenue to develop related drugs to control macrophages in order to ameliorate the inflammatory milieu.

However, the treatment effect of the two strategies mentioned above has limited success.^[^ ^14^ ^]^ This might be attributed to the dynamic and complex nature of the neuroinhibitory microenvironment, composed of two inhibitory components (ROS and neuroinflammation), that can be regenerated via compensatory pathways, resulting in treatment failure.^[^ ^3^, ^10^, ^15^ ^]^ Oxidative stress and inflammation form multiple positive feedback regulatory signaling networks, which dominate the initial post‐injury phase and become landmarks of the extrinsic neural environment. SCI produces ROS through various cellular and enzyme‐mediated signaling pathways. High levels of ROS can easily cause oxidative stress, leading to inflammatory events via multiple mechanisms, such as mediating inflammasome activation, targeting the degradation of I*κ*B, and the translocation of nuclear factor kappa‐B (NF‐*κ*B) to the nucleus and activating inflammation.^[^ ^16^ ^]^ The concomitant persistence of inflammatory cells, especially macrophages, can release tumor necrosis factor (TNF) and induce mitochondrial ROS generation. In turn, this results in the secretion of more ROS by upregulating the expression of iNOS, NADPH oxidase, and other enzymes.^[^ ^15^, ^17^ ^]^ Therefore, modulating a single element (oxidative stress or inflammation) in a complex signaling network consisting of oxidative stress and immunological inflammation is insufficient. Combination techniques may have a synergistic therapeutic impact in this scenario.

**Scheme 1:**
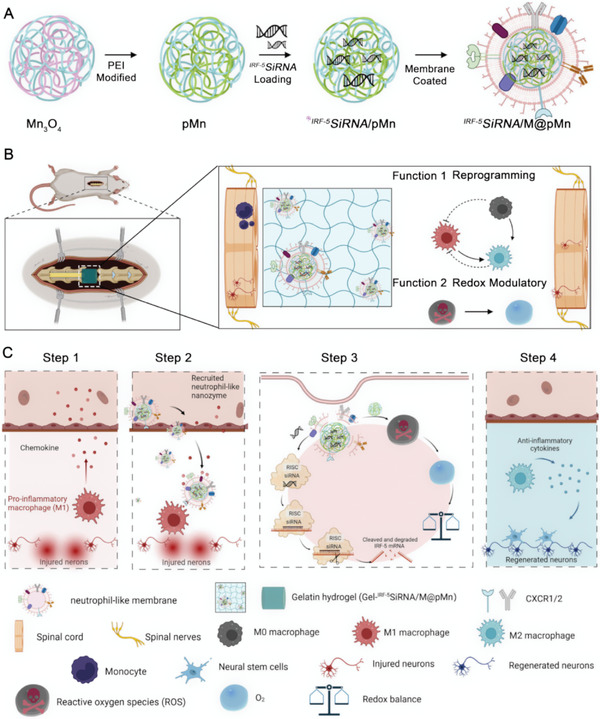
A) Schematic representation of integrated *^IRF‐5^SiRNA*/M@pMn nanozymes. Polyethyleneimine (PEI) was used to modify Mn_3_O_4_ to form pMn. The siRNA was then loaded into the pMn, generating *^IRF‐5^SiRNA*/pMn. The surface of pMn was coated with a neutrophil‐like membrane (*^IRF‐5^SiRNA*/M@pMn). B) Schematic diagram of the multifunctional therapeutic ability of the prepared nanozymes that play the main function; macrophages reprogramming and redox modulatory. The multifunctional nanozyme was blended into a gelatin‐based hydrogel for T9 spinal cord transplantation. C) Diagram showing the recruitment of *^IRF‐5^SiRNA*/M@pMn to inflammatory macrophages.

Herein, we developed a unique combination treatment strategy for SCI that has been proven to be extremely effective in scavenging ROS and reducing inflammation. This strategy involves several mechanisms, as illustrated in **Scheme** **1**A,B. i) First, valence‐engineered nanoflower Mn_3_O_4_ was synthesized, mediating a highly efficient catalytic cascade reaction consisting of multiple antioxidant enzymes (superoxide dismutase, SOD; catalase, CAT; and glutathione peroxidase, GPx) to scavenge ROS.^[^ ^18^ ^]^ ii) For the first time, we designed and screened *^IRF‐5^SiRNA* sequences with strong silencing effects. The “pollen” *^IRF‐5^SiRNA* was loaded into the “nanoflower” modified with the cationic transfection agent polyethyleneimine (*^IRF‐5^SiRNA*/pMn). The subsequent release of *^IRF‐5^SiRNA* may form an RNA‐induced silencing complex (RISC), which could effectively reduce IRF‐5 expression and reprogram M1 macrophages. Inhibiting IRF‐5 expression could effectively inhibit two M0/M2 to M1 macrophage polarization pathways.^[^ ^19^ ^]^ iii) Finally, a neutrophil‐like membrane was coated onto the surface of the *^IRF‐5^SiRNA*/pMn. Nanozymes can be driven by inflammatory factors and recruited to macrophages.^[^ ^20^ ^]^ The effect of cellular uptake was increased by 303%, and M1 macrophage phenotype was reprogrammed successfully (CD206^−^CD86^+^ from 48.5% to 22.4%). In SCI rats, multifunctional integrated nanozyme was blended into a suitable gelatin‐based hydrogel via photo‐cross‐linking for T9 spinal cord transplantation. The synergy between ROS scavenging and inflammation reduction remodeled the extrinsic neuronal environment, promoting the formation of a regenerative landscape characterized by the enhanced proliferation of multiple neuronal phenotypes (motor neurons, interneurons, and sensory neurons), new blood vessel formation, and the recovery of motor function.

## 2 Results and Discussion

### 2.1 Synthesis and Characterization of Valence‐Engineered Nanoflower Mn_3_O_4_

**Figure 1:**
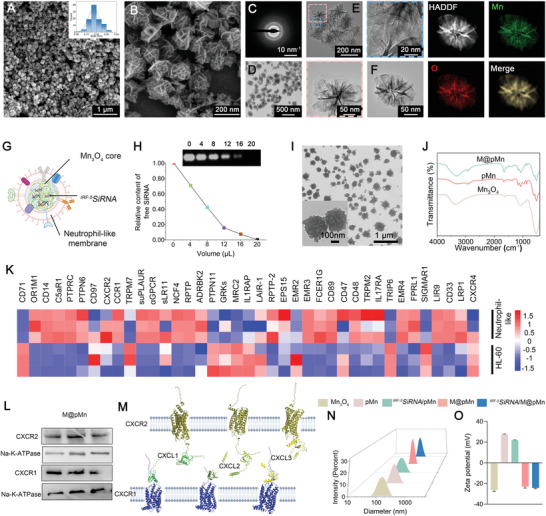
Preparation and characterization of *^IRF‐5^SiRNA*/M@pMn. A,B) SEM, C) SAED patterns, D) TEM, and E,F) HAADF‐STEM images with the corresponding elemental mappings of Mn_3_O_4._ G) Schematic illustration of *^IRF‐5^SiRNA*/M@pMn particles. H) Characterization of pMn/SiRNA nanoparticles using agarose gel electrophoresis at the different volumes of siRNA (0, 4, 8, 12, 16, and 20 µL of 20 µM siRNA) with 2 µL of 1 mg mL^−1^ pMn. I) TEM images of *^IRF‐5^SiRNA*/M@pMn. J) FTIR spectra of Mn_3_O_4_, pMn, and M@pMn nanoparticles. K) Heatmap of membrane protein expression in HL‐60 and neutrophil‐like cells. L) Western blots of CXCR1/2 protein levels in M@pMn. Three lanes in this western blot image reflect triplicate tests. M) Computer simulation image of protein docking between CXCR1/2 and CXCL1/2/3. N) Diameter and O) zeta potential of the different nanoparticles (*n* = 3). Data are presented as mean ± SD. Statistical analysis was performed using a one‐way ANOVA followed by Tukey's post hoc test, *p < 0.05; **p < 0.01; ***p < 0.001; ns, no statistical significance.

The characteristics of the prepared nanoparticle composites were determined using different methods. Scanning electron microscopy (SEM) images (**Figure** **1**A,B) showed distinct nanoflower‐like structures with an average diameter of ≈150 nm (Figure 1A **inset**). The flower‐like structure with a high specific surface area enhanced guest molecule diffusion and exposed more active sites to catalyze reactions while providing more surface binding sites for siRNA loading. The electron diffraction ring of the selected‐area electron diffraction (SAED) patterns confirmed the well‐crystallized nature of Mn_3_O_4_ (Figure 1C).

Transmission electron microscopy (TEM) and high‐resolution transmission electron microscopy (HRTEM) images also indicated the successful synthesis of dispersive Mn_3_O_4_ (Figure 1D,E). The high‐angle annular dark field (HADDF) images clearly showed the structure of the “stems” of the Mn_3_O_4_ nanoflowers (Figure 1F), which were distributed in the brighter areas. The distributions of the Mn and O elements in the EDS elemental mapping were shown in Figure 1F and Figure S1 (Supporting Information).

The catalytic effect of natural SOD was achieved by alternating electron gain and the loss of the metal ions Mn^3+^ (oxidized state) and Mn^2+^ (reduced state).^[^ ^21^ ^]^ Inspired by natural SOD, the valence of Mn was changed from +4 to +3 and +2 using a valence engineering strategy, accomplished by calcining. To get the monodisperse particles of Mn_3_O_4_, precursor MnO_2_ was heated up to 200 °C for 5 h (see more in Supporting Information, experiment section). As Figure S2 (Supporting Information) shows, the high‐resolution X‐ray photoelectron spectroscopy (XPS) spectra of precursor MnO_2_ showed two main spin‐orbit lines in the Mn^4+^ 2p peak, and the atomic percentage of Mn^4+^ was 100% (Figure S2A–C, Supporting Information). Figure S2D–F (Supporting Information) (calcimined Mn_3_O_4_) showed the binding energy peaks of Mn^2+^ 2p_3/2_, Mn^2+^ 2p_1/2_, Mn^3+^ 2p_3/2,_ and Mn^3+^ 2p_3/2_ which were found to be 641.5, 652.9, 642.6, and 654.2 eV, respectively. Quantitative analysis revealed that the atomic percentages of Mn^2+^ and Mn^3+^ were 65.32% and 34.68%, respectively. The shift in the Mn valence state from +4 to +2 and +3 confirmed that precursor MnO_2_ was calcined to form Mn_3_O_4_. Six broad peaks in the X‐ray diffraction (XRD, Figure S3, Supporting Information) pattern were observed at 2*θ* angles of 18.1°, 29.4°, 32.4°, 36.2°, 60.2°, and 65.2°, corresponding to the (101), (112), (103), (211), (224), and (314) planes, respectively (Mn_3_O_4_, JCPDS 80–0382), confirming the XPS results.

### 2.2 Manufacture of Nanozymes Integrating “Pollen” siRNA Delivery

The structure of the nanozyme composite was displayed in Figure 1G, including Mn_3_O_4_ core, “pollen” *^IRF‐5^SiRNA*, and neutrophil‐like membrane. When the PEI/ Mn_3_O_4_ quality ratio was 4:1, the surface ‐NH_2_ group content became saturated, and the total charge approached the greatest positive potential, indicating the optimal binding concentration (Figures S4 and S5, Supporting Information). According to the results of agarose gel electrophoresis, the highest binding quantities between nanoparticles and siRNA were 20 µL (1 mg mL^−1^) and 2 µL (20 µM), respectively (Figure 1H). TEM images of M@pMn revealed increased surface roughness and topography without visible expansion in size compared with Mn_3_O_4_ (Figure 1D) and pMn (Figure S5, Supporting Information), indicating effective cell membrane encapsulation (Figure 1I).

As indicated by the results of the Fourier transform infrared spectroscopy (FTIR) analysis (Figure 1J), the stretching vibration peak of Mn‐O was detected at 524 cm^−1^ in all three samples, without a blue shift or a red shift. The stretching at 1536 and 861 cm^−1^ corresponded to the deformation vibration peaks and rocking vibration peaks of the primary amine N‐H of PEI, respectively. The peak at 1157 cm^−1^ was attributed to the C‐N stretching vibration of PEI, indicating that PEI was successfully modified. The peaks at 1236 and 1543 cm^−1^ in M@pMn were identified as the amide II band from the C‐N stretching or bending vibration of CHN, suggesting effective cell membrane coating. Overall, “pollen” siRNA loading and membrane coating in the integrated nanozymes were successful, as observed directly and indirectly.

After SCI, chemokines trigger the migration of neutrophils, the most accurate chemotactic cells, toward sites of inflammation.^[^ ^20^, ^22^ ^]^ Neutrophil‐based strategies have been used to target neuronal and pulmonary inflammation.^[^ ^23^ ^]^ However, excessive neutrophil activity can result in inflammatory and autoimmune diseases. In experimental research, neutrophils are limited by their short lifespan, undergoing apoptosis within 6–12 h of isolation.^[^ ^20b^ ^]^ The previous report has shown the targeted drug delivery based on the neutrophil‐membrane‐derived nanovesicles can improve current therapies for inflammatory disorders, to overcome these disadvantages, we used neutrophil‐like membranes of human promyelocytic leukemia (HL‐60) cells treated with dimethylsulfoxide (DMSO).^[^ ^24^ ^]^

The absence of organelles renders cell membranes incapable of inducing inflammation. Tandem mass tag (TMT) quantitative proteomic analysis was performed on neutrophils to further investigate their membrane protein composition. As shown in Figure 1K and **Additional Sheet 1**, DMSO‐treated HL‐60 cells exhibited excellent neutrophil chemotactic ability and interaction with the immune system. The proteins CD14, CD89, and CD48 are associated with neutrophil chemotaxis. TMT membrane analysis revealed the presence of chemokine receptors (CXCR2, CXCR4, and CCR1), a family of seven‐pass transmembrane protein receptors connected to guanine nucleotide‐binding proteins (G proteins). Their seven‐pass transmembrane structure effectively prevents protein loss during membrane extraction. Furthermore, western blot analysis confirmed the presence of the receptor proteins CXCR1 and CXCR2, which primarily mediate the interaction of chemokines with M@pMn (Figure 1L; Figure S6, Supporting Information).

Several chemokines, including CXCL1/2/3/4/5/6/7/8, recruit neutrophils to the SCI site. Neutrophil recruitment by CXCL8 is the most extensively studied human analog of rat CXCL1.^[^ ^25^ ^]^ CXCL1/2/3 and CXCL7 are ligands of the CXCR1 and CXCR2 receptors in rats, respectively. CXCR1 and CXCR2 (also known as IL‐8*α* and IL‐8*β* receptors, respectively) are IL‐8 receptors in humans. As a result of receptor and ligand recognition mediated by CXCR1/2 and CXCL1/2/3, the neutrophil‐like membrane nanosystem could be precisely driven by inflammation in both rats and humans (Figure 1M; Figure S7, Supporting Information). Thus, the expression of the CXCL inflammatory chemokine family in rats was examined following SCI (Figure S8; Supporting Information and Additional Sheet 2). The expression of CXCL family members increased to various degrees at different time points after injury, providing a solid in vivo rationale for the chemotactic ability of the targeted nano‐delivery system that causes drug release upon local administration.

The prepared nanoparticles were also analyzed using dynamic light scattering (DLS). As shown in Figure 1N, the average particle sizes of hydrated Mn_3_O_4_, pMn, *^IRF‐5^SiRNA*/pMn, M@pMn, and *^IRF‐5^SiRNA*/M@pMn were 140.6, 171.3, 138.5, 238.0, and 256.3 nm, respectively. The particle size of *^IRF‐5^SiRNA*/pMn slightly decreased compared to pMn, which might be attributed to the loading of negative charges on the nanoparticle surface, resulting in the decreased strength of ionic interactions within the solution. Figure S9 (Supporting Information) showed that the polydispersity indices (PDIs) were all less than 0.3, indicating that these five nanoparticles could remain in solution with outstanding dispersibility. Meanwhile, the zeta potential of Mn_3_O_4_, pMn, *^IRF‐5^SiRNA*/pMn, M@pMn, and *^IRF‐5^SiRNA*/M@pMn were ≈ ‐26.43 ± 1.29 mV, +27.40 ± 0.44 mV, +21.83 ± 0.35 mV, ‐23.00 ± 1.42 mV, and ‐24.33 ± 0.60 mV (Figure 1O). The positive and negative shifts in zeta potential demonstrate that various customized layers were successfully coated on the surface of the nanoparticles.

We manufactured a nano‐delivery system for the extrinsic neural microenvironment after SCI. Even though nanozymes have promising applications in tissue engineering, we further modified nanozymes to endow them, for the first time, with suitable therapeutic potential in SCI. The nanoflower Mn_3_O_4_ exhibited a high specific surface area that could be utilized to load “pollen” siRNA. At the same time, the neutrophil membrane coating increased targeting efficiency and reduced drug intake. Simultaneous protein docking was employed to visualize and investigate the mechanisms underlying neutrophil membrane chemotactic inflammation. We anticipated that bionic nanozymes integrated with targeted siRNA delivery would play a significant role in SCI.

### 2.3 Catalytic Cascade System Comprising Antioxidant Nanozymes

**Figure 2:**
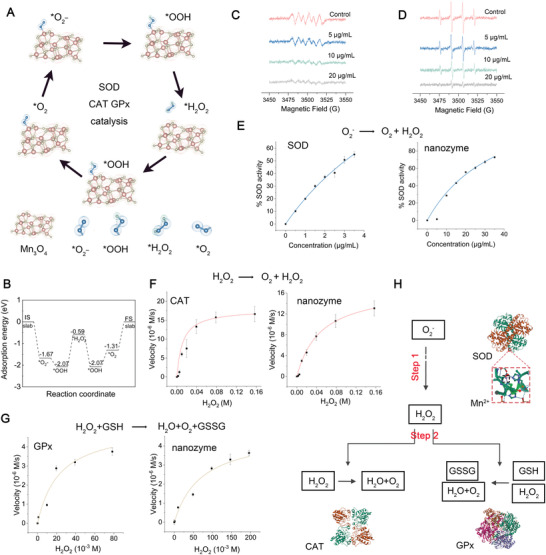
Theoretical investigation and characterization of the multienzyme cascade activities of M@pMn. A) Proposed reaction routes of O_2_^−^ reduction to H_2_O with optimum adsorption configurations. B) Free energy diagram for the O_2_^−^ reduction reaction. EPR for the elimination of C) O_2_^−^ and D) •OH. The spectra were tested at 5 min after mixing in PBS (pH 7.4). E–G) The SOD, CAT, GPx‐like activity of the nanozyme compared to natural SOD, CAT, GPx tested in the same condition (*n* = 3). H) Schematic illustration showing the theoretical nanozyme‐catalyzed cascade reactions. Insert images are the protein structures of the native enzymes. Data are presented as mean ± SD. Statistical analysis was performed using a one‐way ANOVA followed by Tukey's post hoc test, *p < 0.05; **p < 0.01; ***p < 0.001; ns, no statistical significance.

To further explore the origin of the promising catalytic activity and efficiency of Mn_3_O_4_ as an SOD, CAT, and GPx mimic, density functional theory (DFT) calculations were performed for the O_2_^−^ reduction process. The Cambridge Sequential Total Energy Package (CASTEP), which is based on the pseudopotential plane wave (PPW) approach, was used in all DFT computations. **Figure** **2**A shows an energy profile diagram of the most favorable pathway for the reduction of O_2_^−^ using the SOD, CAT, and GPx mimic. The adsorption sites on the Mn_3_O_4_ surface are denoted by asterisks (*). The estimated adsorption energy diagrams demonstrated that the enzymatic cascade mimicked by Mn_3_O_4_ was achievable,^[^ ^26^ ^]^ including the catalysis of H_2_O_2_ generation by O_2_^−^ and the production of O_2_ (Figure 2B). The adsorption energy of O_2_^−^ was lower than that of O_2_, demonstrating that Mn_3_O_4_ more easily adsorbs O_2_^−^ and more easily desorbs O_2_. As O_2_ is easily desorbed, the nanozyme can expose additional binding sites to bind more O_2_^−^, promoting the forward catalytic reaction.

As the composition of ROS was complex and there were no efficient quantitative methods to measure the content of O_2_^−^, electron paramagnetic resonance (EPR) experiments were qualitatively conducted to further monitor the ROS‐scavenging ability of the nanozymes. The xanthine (X)/xanthine oxidase (XO) system was used to produce superoxide anions (O_2_^−^). The characteristic four‐line spectrum peaks (relative intensities of 1:1:1:1) of O_2_^−^ gradually weakened, indicating that the O_2_^−^ scavenging activity of the nanozymes was concentration‐dependent (Figure 2C). Next, the hydroxyl radical‐scavenging effect of the nanozyme was calculated based on its spin spectrum, which showed a 1:2:2:1 intensity pattern. As the Mn concentration increased, the signal intensity decreased (Figure 2D), similar to the result for the O_2_^−^ peaks.

SOD converts superoxide into H_2_O_2_ and O_2_. Superoxide is formed when X reacts with XO, which reduces nitroblue tetrazolium (NBT) to a purple crystal with a maximum absorbance at 565 nm. As shown in Figure 2E (using natural SOD as a positive control), the absorbance decreased remarkably with increasing nanozyme concentration, indicating that the catalytic ability of the SOD mimic was positively correlated with concentration under physiological conditions. As shown in Supplemental Video S1, clear gas bubbles and dissolved O_2_ were observed after adding Mn_3_O_4_ or CAT to tubes containing H_2_O_2_ in PBS (pH 7.4). The CAT‐like activity of the nanozyme was measured based on its ability to decompose H_2_O_2_ into H_2_O and O_2_ (using natural CAT as a positive control). As shown in Figure 2F, the decomposition of H_2_O_2_ as catalyzed by the nanozyme followed Michaelis‐Menten kinetics. A typical GR‐coupled assay was also used to detect the GPx‐mimicking activity of the nanozyme, in which the decrease in NADPH concentration was monitored via spectrophotometry. We studied the catalytic characteristics of the nanozyme, utilizing steady–state kinetics, by varying the H_2_O_2_ concentration (Figure 2G).

The catalytic functionalities and performance of the nanozyme were investigated using typical Michaelis–Menten steady‐state kinetics. Nanozyme assays were carried out using 0.16 and 0.20 m H_2_O_2_ as reactants. The Beer‐Lambert law (formula 1) was used to transform the average starting velocities of absorbance changes into the initial velocities (V0) of O_2_ production or NADPH reduction, which were then plotted against the appropriate concentration and fitted using Michaelis–Menten curves (formula 2). The Michaelis–Menten constant (Km) and maximum velocity (Vmax) were then calculated using a linear double‐reciprocal plot (formula 3), as shown in Supporting Table 4.

(1) A=kbc

(2) $$v_{0}=\frac{V_{\max}\cdot \left[S\right]}{K_{m}+\left[S\right]}$$

(3) $$\frac{1}{v_{o}}=\frac{K_{m}}{V_{\max}}\cdot \frac{1}{\left[S\right]}+\frac{1}{V_{\max}}$$

The *K_m_* (substrate concentration at half *V*_max_) of CAT and GPx of the nanozyme were 0.04813 and 0.07136 m, respectively, which were higher than those of natural CAT (0.01158 m) and GPx (0.02292 m). Oxidative stress occurs at high H_2_O_2_ concentrations (10–1000 µm), which leads to an inflammatory response, growth arrest, and cell death in the central nervous system (CNS). Although SCI would cause tissue acidosis in the secondary injury process, the pH of the injury site remains above 6.6 at 1, 3 and 7 days after spinal cord injury, then the pH gradually becomes neutral. Therefore, Mn_3_O_4_ would induce a limited potential POD catalytic activity in the spinal cord injured site.^[^ ^16^, ^27^ ^]^ Endogenous H_2_O_2_ concentrations at the injured SCI site have frequently been elevated, suggesting that the nanozyme may exert a sufficient therapeutic effect. Moreover, once all active sites of the cascade nanozyme were occupied, the nanozyme could show synergistic ability to catalyze H_2_O_2_ at a maximum velocity of 17.09 M/s (CAT) and 4.701 M/s (GPx), delivering modest yet consistent therapeutic benefits.

Based on the fundamental catalytic properties of the nanozyme, we demonstrated a high‐efficiency cascade reaction without the need for additional enzymes (Figure 2H). The O_2_^−^ produced by oxidative stress is quickly catalyzed by SOD to form H_2_O_2_ (Step 1). Subsequently, the generated H_2_O_2_ is consumed via synergistic catalysis (CAT and GPx, Step 2). The catalytic efficiency of the cascade reaction is significantly enhanced owing to its high local reactant concentration, low intermediate breakdown, and high mass transfer efficiency.^[^ ^28^ ^]^ Meanwhile, the catalytic ability of nanozyme may be slightly decreased due to the loading of siRNA at a specific concentration (Figure S10, Supporting Information). However, siRNA will be released from the surface of Mn_3_O_4_ in cytoplasm, allowing Mn_3_O_4_ to exert its catalytic ability in cascade reactions. Therefore, Mn_3_O_4_, an intelligent artificial “enzyme,” constructs a self‐organized catalytic cascade through a subtle yet sophisticated procedure.

### 2.4 In Vitro Targeting of Inflammatory Macrophages

Although the intake of safe levels of Mn and the outcomes, especially to the nervous system, have been well documented, manganese ions may cause potential risk for nerve damage at high doses. Herein, the CCK‐8 data revealed no significant difference in cytotoxicity among nanoparticles at concentrations of 12.5 and 25 µg mL^−1^ upon incubation with bone marrow‐derived macrophages (BMDMs) and neural stem cells (NSCs) (Figure S11A,B, Supporting Information). Thus, unless otherwise specified, a concentration of 12.5 µg mL^−1^ was used for subsequent in vitro experiments.

**Figure 3:**
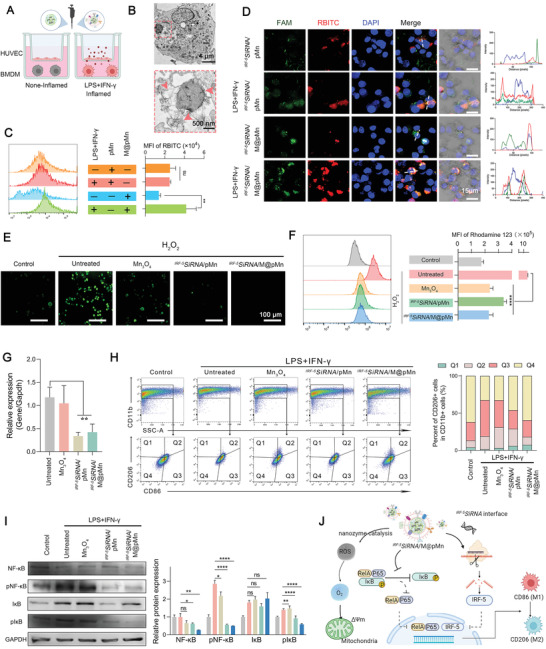
The biological function of *^IRF‐5^SiRNA*/M@pMn on BMDMs includes targeting inflamed macrophages, ROS scavenging, and reducing inflammation. A) Transwell schematic diagram to measure the targeting ability of the nanoparticles. B) Bio‐TEM images of BMDMs cultured with M@pMn (12.5 µg mL^−1^). C) Fluorescence of pMn and M@pMn under non‐inflammatory and inflammatory BMDMs as measured using FACS (*n* = 3). D) Representative confocal images of BMDM incubated with *^FAM‐IRF‐5^SiRNA*/^RBITC^pMn and M@ *^FAM‐IRF‐5^SiRNA*/^RBITC^pMn with or without stimulation using LPS and IFN‐*γ* (Blue, nuclei; red, ^RBITC^pMn; green, ^FAM^siRNA). E) Fluorescence imaging of BMDMs stained with the DCFH‐DA probe. F) MMP quantification and analysis using flow cytometry (*n* = 3). G) mRNA expression levels of IRF‐5 as detected via qPCR (*n* = 5). H) Flow cytometry assay and quantification of BMDM polarization (*n* = 3). M1 and M2 subtypes are distinguished by the presence of CD86 (mostly in Q3) and CD206 (primarily in Q1). I) Western blotting and quantitative analysis of NF‐*κ*B, pNF‐*κ*B, I*κ*B, and pI*κ*B expression in BMDMs (*n* = 3). J) Illustration of antioxidant and anti‐inflammatory processes of *^IRF‐5^SiRNA*/M@pMn treatment in vitro. Data are presented as mean ± SD. Statistical analysis was performed using a one‐way ANOVA followed by Tukey's post hoc test, *p < 0.05; **p < 0.01; ***p < 0.001; ns, no statistical significance.

Due to the interaction of CXCR1/2 (receptors of neutrophils) and chemokines, membrane‐coated NPs could easily be driven to inflammatory M1 macrophages. We established the transwell models to investigate the targeting ability of nanoparticles, where HUVECs mimicked cells that were not expected to be uptaken during SCI (**Figure** **3**A). M@pMn and pMn were internalized into the BMDMs via endocytosis, which were ultimately located in the early endosomal compartments and cytoplasm (Figure 3B; Figure S12A, Supporting Information). This result indicates that cell membranes coated on the surface of pMn did not induce additional endocytic pathways.

As expected, the RBITC mean fluorescence intensity (MFI) of M@pMn‐treated cells assessed via flow cytometry was 303% higher than pMn‐treated cells in inflammatory BMDMs (Figure 3C). The uptake efficiency of M@pMn was lower than that of pMn in non‐inflammatory macrophages, which may be attributed to the enhanced cellular uptake of PEI (positively charged). Meanwhile, the targeting efficiency and siRNA delivery ability of M@pMn were further analyzed using confocal laser scanning microscopy (CLSM) (Figure 3D). The green fluorescence (^FAM^siRNA) colocalized with the red fluorescence (^RBITC^pMn), indicating that M@pMn effectively delivered siRNA drugs into cells. Similar to the results shown in Figure 3B, the intensity curves of DAPI‐ and RBITC‐tagged nanoparticles showed limited overlap, indicating that most nanoparticles were contained in the cytoplasm. The similar target ability can be observed in inflamed microglia (Figure S12B, Supporting Information).

### 2.5 The Nanozyme System Relieves Oxidative Stress and Has Potential Angiogenic Activity In Vitro

After cellular internalization, researchers have been investigating the potential abilities of the nanosystem. Cellular esterases cleaved the DCFH‐DA probe to yield DCFH, which was subsequently oxidized by ROS to produce highly fluorescent 2′, 7′‐dichlorofluorescein (DCF). The gradual decrease in fluorescence intensity (Figure 3E) showed that the nanozyme exhibited good catalytic performance in cells. High levels of oxidative stress can also cause depolarization of the mitochondrial membrane potential (MMP). Our results showed that Mn_3_O_4_, *^IRF‐5^SiRNA*/pMn, and *^IRF‐5^SiRNA*/M@pMn restored MMP depolarization via ROS scavenging (Figure 3F), which also prevented the imbalance in membrane potential, apoptosis, and autophagy. This conclusion was also supported by the cell viability test (Figure S13, Supporting Information).

SCI‐induced blood vessel loss and the weakening of the blood‐spinal cord barrier could lead to inflammation, ischemia, and consequently, total spinal cord nerve tissue damage.^[^ ^29^ ^]^ However, a prolonged oxygen release mechanism that also significantly scavenges ROS may hasten wound repair. Sustained O_2_ release has been shown to stimulate endothelial tube formation and angiogenesis.^[^ ^30^ ^]^ Here, the nanozymes showed a synergistic ability to catalyze high levels of endogenous ROS to O_2_. Based on the above findings, we developed an in vitro angiogenesis model using HUVECs to verify this hypothesis. Figure S14 (Supporting Information) showed angiogenesis failure in the H_2_O_2_ treatment group, but the addition of nanozymes effectively reversed this effect. However, increased angiogenesis was not observed in the NP‐treated group compared with the other groups. This might be attributed to the in vitro model, wherein the 5% CO_2_ atmosphere in the cell incubator balanced the produced O_2_. Hence, we would further elucidate the function of oxygen production at the injury site in vascular recovery in vivo.

### 2.6 Gene Silencing Effectiveness and Reversal of The Inflammatory Phenotype of Macrophages to Fight Inflammation

The coding sequence of IRF‐5 (Gene Number: 296953) was retrieved from the National Center for Biotechnology Information database (NCBI, Supporting Table 5). Its antisense sequence was constructed by screening the potential silencing sites, and two TT bases were added as overhangs at its tail (Figure S15A, Supporting Information). Under the assumption of assuring transfection efficiency (Figure S15B, Supporting Information), the silencing efficiencies of the three proposed siRNA sequences were ‐47.57%, 28.94%, and 76.86%, respectively. *^IRF‐5^SiRNA‐818* was used in subsequent experiments; *^IRF‐5^SiRNA* was used as a substitute for *^IRF‐5^SiRNA‐818* unless otherwise specified (Figure S15C, Supporting Information).

The mRNA expression of IRF‐5 decreased sharply in the *^IRF‐5^SiRNA*/pMn and *^IRF‐5^SiRNA*/M@pMn groups, suggesting that the siRNA can form RISC in macrophages to reduce target gene expression (Figure 3G). Fluorescence‐activated cell sorting (FACS) results indicated that the proportion of macrophage polarization changed in the treated groups (Figure 3H). Within the CD11b^+^ cell population, the proportion of CD206^+^CD86^−^ macrophages increased slightly in the *^IRF‐5^SiRNA*/pMn‐ (6.02%) and *^IRF‐5^SiRNA*/M@pMn‐treated (7.35%) groups compared to the untreated groups (1.48%). Also, by comparing Mn_3_O_4_‐treated (3.15%) and *^IRF‐5^SiRNA*/pMn‐treated (6.02%) groups, the combined effect with ROS scavenging and anti‐inflammatory showed a more effective role. In contrast, the proportion of CD206^−^CD86^+^ macrophages were significantly lower in the *^IRF‐5^SiRNA*/pMn‐ (25.0%) and *^IRF‐5^SiRNA*/M@pMn‐treated (22.4%) groups than in the untreated groups (48.5%), indicating that the NPs could successfully reprogram inflammatory macrophages toward reparative phenotypes.

During CNS inflammation, I*κ*B proteins bind to NF‐*κ*B, preventing their nuclear translocation and binding to DNA. Lipopolysaccharides (LPS) and IFN‐*γ* activate the I*κ*B‐kinase complex, which is response for phosphorylating I*κ*B molecules at certain regulatory amino acid residues. To this end, I*κ*B was targeted for degradation, allowing NF‐*κ*B to translocate to the nucleus and activate its target genes. However, pI*κ*B and pNF‐*κ*B levels were significantly decreased after treatment with NPs (Figure 3I), indicating that the NPs could effectively block the NF‐*κ*B inflammatory signaling pathway and reduce the inflammatory feedback of macrophages.

As shown in Figure 3J, *^IRF‐5^SiRNA*/M@pMn particles inhibited ROS generation, which maintained MMP homeostasis and I*κ*B degradation, reduced downstream NF‐*κ*B signaling, and prevented its translocation to the nucleus and binding to inflammation‐related genes. Silencing resulted in a decrease in IRF‐5 expression and a reversal of macrophage pro‐inflammatory phenotype. This evidence encouraged us to proceed with using this nanosystem as a therapy in vivo.

### 2.7 Characterization and Biocompatibility Analysis of Gelatin Hydrogels Blended with Nanozymes

**Figure 4:**
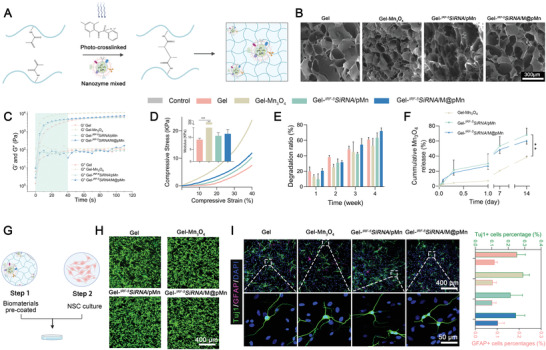
Characterization and biocompatibility analysis of gelatin hydrogels matched to native spinal cord healing processes. A) Schematic diagram of the prepared nanozymes mixed with hydrogels. B) SEM images of the different hydrogels. C) Cross‐linking of the prepolymer solution was monitored via rheological analyses. D) Representative stress−strain curves of the different hydrogels. Insert images represent the compressive modulus (*n* = 3). E) Degradation ratio of the hydrogel after incubation in saline for 1, 2, 3, and 4 weeks (*n* = 3). F) Mn_3_O_4_ sustained release behavior of the hydrogels (*n* = 4). G) Cell adhesion on the surface of the gelatin hydrogel. H) NSCs showed high cell viability even after 5 d of culture (green; live cells, red; dead cells). I) Representative images and quantification analysis (*n* = 6) of Tuj‐1 (for neurons) and GFAP (for astrocytes) expression, showing the growth of NSCs on the gelatin hydrogel after 7 d of culture. Data are presented as mean ± SD. Statistical analysis was performed using a one‐way ANOVA followed by Tukey's post hoc test, *p < 0.05; **p < 0.01; ***p < 0.001; ns, no statistical significance.

With the inclusion of cell attachment and matrix metalloproteinase‐sensitive peptide motifs, GelMA hydrogels could closely mirror several important features of the native extracellular matrix, allowing cells to proliferate in GelMA‐based scaffolds.^[^ ^31^ ^]^ Recent research has proven that GelMA‐based hydrogels are suitable for implant applications in the spinal cord.^[^ ^32^ ^]^ GelMA coupled with nanozymes underwent photoinitiated radical polymerization in the presence of a photoinitiator (lithium phenyl [2,4,6‐trimethylbenzoyl] phosphinate, LAP) under UV light exposure to generate covalently cross‐linked hydrogels for implantation at the SCI site, as shown in **Figure** **4**A and Figure S16 (Supporting Information). The stretching vibrations of the unsaturated —C=C— double bond (1633 cm^−1^) and the stretching vibration peaks of —C=C—H (3306 cm^−1^) were lost after photo‐crosslinking, implying that —C=C— is involved in photo‐cross‐linking in the GelMA (Figure S17, Supporting Information). SEM was used to explore the morphological characteristics of the hydrogels. The results revealed an interconnected porous network in the hydrogel with pore diameters of ≈150 µm, demonstrating their capacity as a cell living space, supporting nutrition and waste movement. The addition of nanozymes to the gelatin‐based hydrogel substantially reduced the pore size of the nanocomposite hydrogels, which may be attributed to an increase in the cross‐linking density generated by the nanoparticles (Figure 4B).^[^ ^33^ ^]^ This conclusion was confirmed by the subsequent rheological and compressive modulus tests. The gelatin‐based hydrogels were strengthened by the addition of a nanoparticulate substance that served as a cross‐linking epicenter. Rheological tests demonstrated that the hydrogel could form a covalently crosslinked network within ≈40 s, which could be employed for further applications in vivo (Figure 4C). The compression modulus showed mechanical properties similar to those of the natural spinal cord (<10 kPa) (Figure 4D).

The mechanical properties of the implant materials must adapt to the development of new tissues, making them appropriate for cell proliferation and neural expansion. Biodegradable materials should minimize prolonged inflammatory reactions, restrict blockage, and stimulate nerve development by enabling replacement with renewing cells and their corresponding matrices. As a result, the Gel, Gel‐*^IRF‐5^SiRNA*/pMn, and Gel‐*^IRF‐5^SiRNA*/M@pMn groups degraded by over 60% after 4 weeks, which matched the process of spinal cord repair in SCI models (Figure 4E). The drug release ability of the NPs also adapted to the degradation of the gelatin hydrogel, and the contents of the supernatant released to the solution after 14 days were 38.54%, 68.21%, and 60.38%, respectively (Figure 4F). After the composite hydrogel degrading, M@pMn was collected by centrifugation and purification, and the complete membrane structure was observed by TEM (Figure S18, Supporting Information).

The fabricated porous hydrogels mimicking the extracellular matrix exhibited mechanical properties that matched the native spinal cord and appropriate degradation ability adapted to the regeneration of spinal cord tissue. Hence, the prepared gelatin‐based hydrogels could be candidate natural materials for the recovery of spinal cord injuries. The ability of cells to attach, grow, and differentiate inside functional hydrogels is of fundamental importance for neuronal regeneration in the spinal cord. In this study, cell viability and differentiation were determined by identifying the cells that adhered to the surfaces of hydrogels made from different nanoparticles (Figure 4G, Gel, Gel‐Mn_3_O_4_, Gel‐*^IRF‐5^SiRNA*/pMn, and Gel‐*^IRF‐5^SiRNA*/M@pMn). Primary neural stem cells (NSCs), which have similar properties in vitro, were used in this study for neuronal regeneration. Figure 4H,I demonstrate the innate biocompatibility of the different hydrogels, while the biomaterials did not affect the direction of differentiation of neurons and astrocytes. These results suggest that the prepared hydrogels mixed with different nanoparticles showed excellent biocompatibility in vitro and could be utilized as implant materials in a rat SCI model.

### 2.8 The Gel‐*^IRF‐5^SiRNA*/M@pMn System Reduced Oxidative Stress and Promoted M2 Macrophage Polarization In Vivo

**Figure 5:**
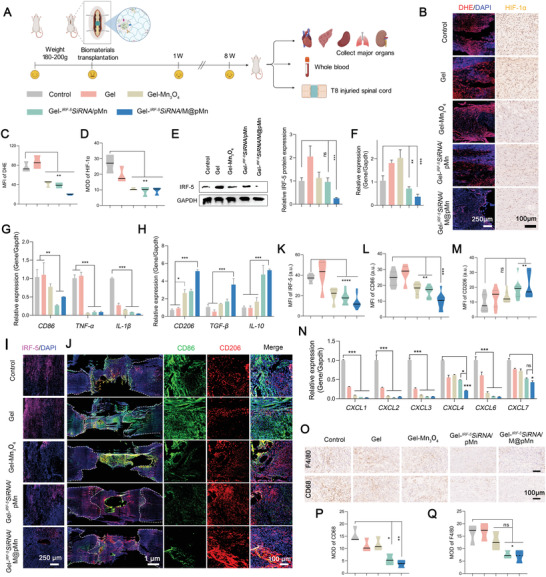
Evaluation of short‐term treatment effect in animal models. A) Treatment regime in rats. B) Representative fluorescence staining images of DHE and immunohistochemical images of HIF‐1*α* in the injured spinal cord tissues and quantitative analysis of C) DHE and D) HIF‐1*α* levels (*n* = 6). E) Western blot (*n* = 3) and quantification, and F) qPCR of IRF‐5 expression levels (*n* = 5). qPCR analysis of the expression levels of G) M1 markers (*CD86*, *TNF‐α*, *IL‐1β*) and H) M2 markers (*CD206, TGF‐β, IL‐10*) (*n* = 5). I) IRF‐5‐, J) CD86‐ (M1, green), and CD206‐ (M2, red) positive cells at the lesion site of SCI model rats. Quantification of K) IRF‐5, L) CD86, and M) CD206 expression (*n* = 6). N) qPCR to detect *CXCL1, 2, 3, 4, 6*, and *7* expression levels (*n* = 5). O) Representative immunohistochemical images and quantification analysis of F4/80 and CD68‐positive cells at the spinal cord injury site (*n* = 6). Data are presented as mean ± SD. Statistical analysis was performed using a one‐way ANOVA followed by Tukey's post hoc test, *p < 0.05; **p < 0.01; ***p < 0.001; ns, no statistical significance.

Microglia/macrophages at the SCI site reached their highest numbers at 5–7 days.^[^ ^15^ ^]^ The protocol for rat SCI model establishment is shown in **Figure** **5**A and Figure S19 (Supporting Information). Short‐term acute inflammatory responses and long‐term motor function recovery were observed at 7 and 8 weeks, respectively. Electron accumulation during hypoxia leads to the formation of O_2_^−^ in the mitochondria, thereby stimulating the production of hypoxia‐inducible factor HIF‐1*α*. Dihydroethidium (DHE) is a commonly used fluorescent probe to detect superoxide anion levels in the respiratory burst of phagocytes. Figure 5B–D show the decreased MFI of DHE and mean optical density (MOD) of HIF‐1*α*, indicating that the nanozyme could catalyze the conversion of toxic ROS to non‐toxic O_2_, effectively reducing oxidative stress and relieving local hypoxia.

Figure 5E,F show that the IRF‐5 protein and mRNA expression decreased by 73.08% and 63.10%, respectively, which was attributed to the silencing efficiency of *^IRF‐5Si^RNA*. The increased IRF‐5 expression in the Gel group might be related to the acute inflammation generated by implanting gelatin hydrogels. The decrease in the protein expression of IRF‐5 led to macrophage reprogramming, which was confirmed by the changes in the gene expression levels of *CD86* and *CD206* via FACS analysis (Figure 5G,H). Changes in macrophage polarization led to the reduced expression of pro‐inflammatory factors (*TNF‐α* and *IL‐1β*) and a significant increase in the expression of anti‐inflammatory factors (*TGF‐β* and *IL‐10*). The decreased expression of IRF‐5 was also clarified via CLSM (Figure 5I,K). Figure 5J,L,M showed that the ratio of CD206/CD86 increased, suggesting the increased proportion of reparative M2 macrophages at the injury site. The above data also showed that the expression level of IRF‐5 and inflammatory factors (*TNF‐α* and *IL‐1β*) was significantly reduced in Gel‐*^IRF‐5^SiRNA*/M@pMn group, indicating that target delivery efficiency was enhanced by the neutrophil‐like membrane coating. Potentially novel strategies of neuronal protection during SCI treatment target inflammation via immunomodulation and the activation of reparative M2 macrophages using pharmacological and transplant interventions.^[^ ^2^ ^]^

As previously determined (Figure S8, Supporting Information), the reduced expression of CXCL family proteins (*CXCL1, 2, 3, 4, 6*, and *7*) confirmed the attenuation of neutrophil infiltration (Figure 5N), partly explaining the inflammation‐modulating capacity. In addition, the decreased expression of inflammatory markers at the SCI site, including CD68 and F4/80, indicate a progressive reduction in inflammatory macrophage activation, expansion, and infiltration (Figure 5O–Q).

Liver and kidney retain Mn more than other tissues and the liver plays a critical role in this process and is the main organ responsible for Mn excretion. Therefore, the potential cytotoxicity of manganese oxidase was further analyzed. As for the blood cell analyses, all groups exhibited negligible changes in the levels of WBCs (white blood cells), RBCs (red blood cells), PLT (platelets), and HGB (hemoglobin), demonstrating that the nanosystem did not induce obvious damage to the blood (Figure S20A, Supporting Information). Levels of the biochemical parameters alanine aminotransferase (ALT), alkaline phosphatase (ALP), aspartate aminotransferase (AST), and UREA (creatinine) were maintained, indicating that the material did not cause significant hematological toxicity (Figure S20B, Supporting Information). Representative histological analysis of H&E‐stained rat organs revealed no noticeable signals of major organ damage, suggesting the satisfactory biocompatibility of the implant hydrogels in vivo (Figure S21, Supporting Information).

Short‐term in vivo animal experiments confirmed that the Mn_3_O_4_ nanozyme loaded with *^IRF‐5^SiRNA* effectively reduced oxidative damage and relieved hypoxia, promoted the phenotypic expression of M2 macrophages, reduced the infiltration of neutrophils and macrophages, and weakened the immune storm during SCI. Furthermore, when the Gel‐Mn_3_O_4_ and Gel‐*^IRF‐5^SiRNA*/pMn groups were compared, ROS scavenging and reduced inflammation simultaneously resulted in a greatly improved anti‐inflammatory and anti‐oxidant effect, avoiding the generation of compensatory pathways between inflammation and ROS, indicating the superiority of combined therapy. We expected the extrinsic neural environment to be remodeled by the Gel‐*^IRF‐5^SiRNA*/M@pMn at the SCI site to encourage neuronal regeneration by allowing endogenous neurons to survive, migrate, differentiate, and improve neovascularization until the recovery of motor function in SCI model rats.

### 2.9 Accelerating Neuronal Formation and Enhancing Long‐Term Motor Function Recovery

**Figure 6:**
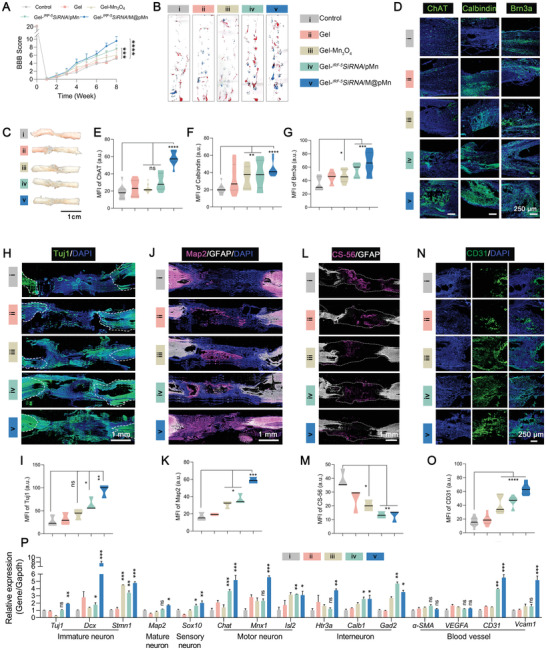
The functional hydrogel induced excellent recovery in long‐term animal experiments. A) BBB scores of rats from surgery to eight weeks post‐injury (*n* = 8). B) Examination of hind limb motor function recovery in rats using typical footprints (forepaw, blue ink; hind paw, red ink.) C) Representative spinal cord images after SCI repair. D) Representative immunofluorescence images after staining for ChAT, Calbindin, and Brn3a and quantification of E) ChAT, F) Calbindin, and G) Brn3a staining (*n* = 6). The neuronal differentiation profiles of H) Tuj1, I) Map2, L) CS56, and N) CD31, and their corresponding quantitative analyses J), K), M), O), respectively in the lesion area of SCI rats (*n* = 3). P) qPCR analysis of the expression of neuron and blood vessel markers (*n* = 5). Data are presented as mean ± SD. Statistical analysis was performed using a one‐way ANOVA followed by Tukey's post hoc test, *p < 0.05; **p < 0.01; ***p < 0.001; ns, no statistical significance.

Rats in the Gel‐*^IRF‐5^SiRNA*/M@pMn group (BBB score: 9.57 ± 1.13) exhibited significant improvement compared with rats in other groups (BBB scores of the Control: 5.14 ± 1.46, Gel: 5.43 ± 2.07, Gel‐Mn_3_O_4_: 6.00 ± 1.15, and Gel‐*^IRF‐5^SiRNA*/pMn: 7.57 ± 0.98; **Figure** **6**A). Rats in the treatment group (Gel‐*^IRF‐5^SiRNA*/M@pMn) showed clearer footprints (Figure 6B), and the inclined plane test suggested an improvement in rat grip (Figure S22, Supporting Information). The recovery of the rats’ motor function was also demonstrated (Supporting Video 2). The Gel‐*^IRF‐5^SiRNA*/pMn group elicited weight‐bearing movement of the palmar surface of the paw, even in the absence of coordinated movement in the front and rear limbs. The above results demonstrated the recovery of rat motor function, which was also confirmed by the restored connections in the anatomical picture of the injured spinal cord shown in Figure 6C.

Diversification of the neuronal subtypes is required for the formation of functional neural circuits. Neurons in the spinal cord were classified according to their function as sensory, motor, or interneurons. However, the molecular events underlying neuronal diversity after spinal cord injury remain largely unexplored. According to the time course of SCI, several neuronal subtypes of neurons were analyzed as evaluation indicators after treatment.

Immunofluorescence images of tissue cryosections were employed to verify protein expression and colocalization at the injury site. Figure 6D–G showed that the expression of ChAT (motor neuron marker), Calbindin (interneuron marker), and Brn3a (sensory neuron marker) were significantly increased. Likewise, substantially higher numbers of Tuj‐1‐positive (newborn neurons) and Map2‐positive (mature neurons) cells were also observed in the lesion area of rats in the Gel‐*^IRF‐5^SiRNA*/M@pMn group than in other groups (Figure 6H–K), which could be extensively detected throughout the entire lesion area.

Activated macrophages are involved in scar formation. The inhibition of scar formation was demonstrated by staining with CS‐56, which specifically recognizes the glycosaminoglycan (GAG) component of the natural chondroitin sulfate proteoglycan (CSPG). The Gel‐*^IRF‐5^SiRNA*/M@pMn group had significantly lower CSPG‐positive staining at the lesion site than the other groups (Figure 6L,M), indicating that inflammation regulation during the acute injury phase of SCI can effectively reduce scarring and minimize the physical barriers to nerve growth and angiogenesis. We also quantified the expression densities of CD31 (also named platelet endothelial cell adhesion molecule) in the rats in the treatment groups with spinal cord‐injured tissues. Enhanced CD31 expression in these groups revealed that simultaneous ROS scavenging and continuous oxygenation strongly promoted angiogenesis (Figure 6N,O).

Based on the above protein expression verification, gene expression at the mRNA level in the injured area was determined via qPCR (Figure 6P). The results showed that the expressions of the markers of immature neurons (*Tuj1*, *Dcx*, and *Stmn1*), mature neurons (*Map2*), sensory neurons (*Sox10*), motor neurons (*Chat*, *Mnx1*, and *Isl2*), and interneurons (*Htr3a*, *Calb1*, and *Gad2*) were significantly increased. Surprisingly, we also observed the formation of blood vessels (*CD31 and Vcam‐1*). However, the gene and protein expression of VEGFA showed an unexpected downward trend (Figure S23, Supporting Information), which may trigger the HIF‐1*α*‐coupled VEGFA signaling pathway.^[^ ^34^ ^]^

Similar to the results of the analysis of blood cell counts and biochemical parameters in the short‐term treatment experiments, the implanted material did not cause obvious toxicity in long‐term experiments (Figure S24A,B, Supporting Information), which was also confirmed by the histological examination of H&E‐stained organs (Figure S25, Supporting Information). The PLT count in the Gel‐*^IRF‐5^SiRNA*/pMn treatment group was slightly lower than in the other groups. However, the levels of biochemical parameters in the Gel‐*^IRF‐5^SiRNA*/M@pMn group were similar to those in the control group, indicating the safety of the target nanozyme.

## 3 Conclusion

Over the past decades, previous strategies significantly improved long‐distance axonal regeneration and efficient connectivity of functional neurons by remodeling the microenvironment after SCI, such as using dual‐functional scaffold‐scavenged anionic DAMPs, exhibiting sustained release of IL‐10 to reduce the pro‐inflammatory responses of macrophages and microglia,^[^ ^35^ ^]^ and designing MMP‐responsive bionic mechanical and conductive hydrogels to restore the spinal cord biophysical microenvironment.^[^ ^36^ ^]^ Hence, remodeling the extrinsic neural environment after SCI has become an important therapeutic target.

Nanozymes appear to have surprising biological effects because they imitate enzymatic function while causing distinct inflammatory responses in immune cells in various disease environments.^[^ ^37^ ^]^ Overall, the anti‐inflammatory effect of antioxidant enzymes is caused by eliminating high ROS levels, which indirectly cause the polarization of macrophages toward the M2 phenotype by reducing inflammation or the nuclear translocation of NF‐*κ*B. Although the use of nanozymes in SCI has not been reported, we believe that ROS scavenging by nanozymes helps promote macrophage polarization toward the reparative phenotype, which is compatible with our findings (Figure 5). However, due to the complicated tissue microenvironment in SCI, we reduced the expression of IRF‐5 as an essential target for macrophage reprogramming to avoid the development of compensatory pathways in which inflammation and ROS are exacerbated.

In this study, we constructed multifunctional nanozymes integrated with siRNA technology, which exhibited crucial synergistic anti‐inflammatory and antioxidant effects in the pathological neuronal environment of SCI in vitro and in vivo. A suitable extrinsic neural environment induced the differentiation of various neuronal subtypes, leading to a reduction in inflammation, which also reduces scar formation (Figure 6). Surprisingly, nanozymes showed synergistic ability in catalyzing the conversion of high levels of ROS to O_2_ without eliciting biological toxicity. In addition, the sustained release of O_2_ stimulates endothelial tube formation and angiogenesis. High levels of ROS and inflammatory as two major inhibitory factors have steadily broadened our understanding of the autoimmune regulation system and the injured microenvironment in numerous damage scenarios. Given that many physiologies and diseases are accompanied by ROS and inflammation^[^ ^3^, ^16^, ^19^, ^20^ ^]^, the strategy of multifunctional integrated nanozymes we developed may have broad applications in a range of disease models, including the neurological, cardiovascular, and immunological systems, skeletal muscle and metabolic control, and aging.

## 4 Experimental Section

The experimental details are provided in the Supporting Information.

### Animals

All animal experiments conformed to the National Institutes of Health guidelines and protocols were approved by the committee of Suzhou Institute of Nano‐Tech and Nano‐Bionics, the Chinese Academy of Sciences (Approval number: SINANO/EC/2022–070).

### Statistical Analysis

Quantitative data were presented as mean ± standard deviation, and obtained from at least triple independent experiments. One‐way ANOVA was used to assess the statistical difference between distinct sets of data followed by Tukey's post hoc test, and *p* < 0.05, *p* < 0.01, and *p* < 0.001 were considered significant, highly significant and extremely significant respectively and indicated separately with *, ** and ***. Statistical significance between groups was determined using SPSS (Statistical Product and Service Solutions).

## Conflict of Interest

The authors declare no conflict of interest.

## Supporting information

Supporting Information

Supplemental Video 1

Supplemental Video 2

Supporting Information

Supporting Information

## Data Availability

The data that support the findings of this study are available from the corresponding author upon reasonable request.
