# Supplementary material for: Multifunctional Integrated Nanozymes Facilitate Spinal Cord Regeneration by Remodeling the Extrinsic Neural Environment
Source: Adv Sci (Weinh). 2023 Jan 16;10(7):2205997. doi: 10.1002/advs.202205997 (PMC9982579; doi:10.1002/advs.202205997)
Supplement: Supplementary file 1 — Supporting Information [file ADVS-10-2205997-s001.pdf]

ADVANCED SCIENCE
Open Access

Supporting Information

for *Adv. Sci.*, DOI 10.1002/advs.202205997

Multifunctional Integrated Nanozymes Facilitate Spinal Cord Regeneration by Remodeling the Extrinsic Neural Environment

Tiandi Xiong, Keni Yang, Tongtong Zhao, Haitao Zhao, Xu Gao, Zhifeng You, Caixia Fan, Xinyi Kang, Wen Yang, Yan Zhuang, Yanyan Chen and Jianwu Dai**

Supporting Information

Multifunctional Integrated Nanozymes Facilitate Spinal Cord Regeneration By Remodeling the Extrinsic Neural Environment

Tiandi Xiong, Keni Yang, Tongtong Zhao, Haitao Zhao, Xu Gao, Zhifeng You, Caixia Fan, Xinyi Kang, Wen Yang, Yan Zhuang, Yanyan Chen, and Jianwu Dai**

T. Xiong, T. Zhao, W. Yang, Y. Zhuang
School of Nano Technology and Nano Bionics, University of Science and Technology of China, Hefei 230026, China

T. Xiong, Dr. K. Yang, T. Zhao, H. Zhao, X. Gao, Dr. Z. You, Dr. C. Fan, X. Kang, W. Yang, Y. Zhuang, Prof. Y. Chen, and Prof. J. Dai
Key Laboratory for Nano-Bio Interface Research, Division of Nanobiomedicine, Suzhou Institute of Nano-Tech and Nano-Bionics, Chinese Academy of Sciences, Suzhou 215123, China

Prof. J. Dai
State Key Laboratory of Molecular Development Biology, Institute of Genetics and Developmental Biology, Chinese Academy of Sciences, Beijing 100101, China

Prof. Y. Chen
Key Laboratory for Nano-Bio Interface Research, Division of Nanobiomedicine, Suzhou Institute of Nano-Tech and Nano-Bionics, Chinese Academy of Sciences, Suzhou 215123, China
E-mail: yychen2006@sinano.ac.cn

Prof. J. Dai

State Key Laboratory of Molecular Development Biology, Institute of Genetics and Developmental Biology, Chinese Academy of Sciences, Beijing 100101, China
E-mail: jwdai@genetics.ac.cn

Experimental Section

Regent

$KMnO_4$ was purchased from Sinopharm Group Chemical Reagent Co., Ltd. Methacrylate Gelatin (GelMA, the degree of esterification of MA was ~65%), Polyethyleneimine (PEI), and Oleic acid (OA) were obtained from Aladdin Industrial Co., Ltd. (Shanghai, China). SiRNA was designed and synthesized from GenePharma Co., Ltd (Shanghai, China). Rhodamine 123 and and Rhodamine B isothiocyanate (RBITC) were purchased from Sigma Aldrich (St. Louis, MO, USA). (Lithium Phenyl (2,4,6-Trimethylbenzoyl) phosphinate, LAP) was obtained from Tokyo Chemical Industry Co. Ltd. (Tokyo, Japan). All the reagents and chemicals were of analytical reagent grade unless otherwise stated.

Synthesis of Mn_3O_4

Mn_3O_4 was manufactured using a slightly modified version of the previously reported[1]. In brief, $KMnO_4$ (1.0 g) was dissolved in ultrapure water (500 mL) and rapidly swirled for 30 minutes and then added oleic acid (10 mL) to the reaction mixture and stirred for 24 hours at 28 °C water bath. The as-synthesized solution was centrifuged at 12,000 rpm for 20 min to collect the black precipitate. To get MnO_2 nanoparticles, the precipitate was washed with ultrapure water and anhydrous ethanol until the supernatant was clear, then dried in a vacuum at 45°C overnight. To get the monodisperse particles of Mn_3O_4, MnO_2 was heated in a muffle furnace (Jinghong, Shanghai) up to 200 °C for 5 h.

Preparation of PEI modified Mn_3O_4

The cross-linking of Mn_3O_4 and polyethyleneimine (PEI) was carried out by electrostatic conjugation. The reaction was carried out according to the mass ratio of Mn_3O_4: PEI=1:1, 1:2, 1:3, 1:4, 1:5, Mn_3O_4 (1mg/mL) was pre-dispersed by an ultrasonic crusher at the condition of Φ6 operating rod, 20% power, ultrasonic for 20 min (work for 4 s, stop for 4 s). The corresponding quality of PEI was then added and

shaken to react for 12 h at 220 rpm, 37 °C in the shaker. The precipitation was collected at 10,000 rpm for 20 min. After washing with PBS (pH 7.4), the product was dispersed in PBS (pH 7.4), and stored at 4°C for future use.

SiRNA loading

SiRNA solution (20 μM, 2 μL) was added to PEI-Mn (1mg/mL) in various volumes (0, 4, 8, 12, 16, 20 μL). And the solution was gently mixed gently for 15 min at 37 °C water bath, allowing adequate binding of SiRNA molecules to the pMn. The binding capacity of SiRNA to pMn was determined using agarose gel electrophoresis. After centrifugation, the solution was placed into a 2% agarose gel for electrophoresis in TBE buffer at a constant voltage of 45 V for 20 minutes. To assess loading capacity, SiRNA was stained with goldview (Yeasen, China).

Membrane coated

Cell Membrane Fragment Extraction: The neutrophil-like cell membrane was obtained according to freeze-thaw cycles and physical disruption methods[2]. The cells collected were frozen and thawed three times in liquid nitrogen. Then the obtained cells were placed in an ultrasonic bath for 5 min before being centrifuged (700 g, 10 min, 4 °C) to obtain supernatant and subsequently centrifuged (14,000 g, 30 min) to obtain the membrane fragments. The protein content was then quantified using the traditional BCA (Bicinchoninic Acid Assay). Finally, the membrane fragments were lyophilized and stored at 80 °C for future use.

Cell membrane and nanoparticles were added to the solution at a mass ratio of 1:1 to mix under an ultrasonic bath for another 30 min and then physically extruded 21 times by a mini extruder (Avanti, USA) through porous polycarbonate membranes (1μm and 400 nm). Finally, the obtained nanoparticles were centrifuged and washed with PBS for three times before being quantitatively evaluated using BCA protein quantification.

TMT protein analysis

Quantitative proteomics analysis of membrane protein was conducted by TMT (Tandem Mass Tag) technology. TMT quantitative proteomics analysis mainly includes the following 6 steps: total protein extraction, protein quality test, TMT labeling of peptides, separation of fractions, liquid chromatography−tandem mass spectrometry (LC-MS/MS) analysis and data analysis. All raw data were analyzed in the database homo_sapiens_uniprot_2022_1_27.fasta.fasta (203746 sequences) by using the library search software Proteome Discoverer 2.4.

Protein docking and visualization

Binding crystals of IL-8 and CXCR1/CXCR2 which have been resolved[3], were directly downloaded from the Protein Data Bank (PDB) database and visualized in PyMol Stereo 3D Zalman (CXCR1/IL-8, PDB;1ILQ, CXCR2/IL-8, PDB ID; 6LFO). Protein mimetic dockings of CXCR1/2 and CXCL1/2/3 were accomplished via ClusPro Protein-Protein docking[4].

Characterization methods

The as-prepared nanoparticles were morphologically characterized with high-angle annular dark field scanning TEM (HITACHI, Japan), high-resolution transmission electron microscopy (FEI Talos F200X G2, USA) and SEM (HITACHI, Japan). The crystal structure of the Mn_3O_4 was analyzed using an X-ray diffractometry (XRD, Rigaku SmartLab SE, Japan). The FTIR spectra of the nanoparticles were recorded using Fourier transform infrared spectroscopy (FTIR, Thermo Scientific Nicolet iS5, USA). The XPS was measured using a Thermo Scientific K-Alpha spectrometer (USA). Dynamic laser scattering (DLS) was used to determine zeta potential and hydrated diameter on Malvern Zetasizer Nanoseries (Malvern, USA).

Density Functional Theory (DFT) theoretical calculation

The Cambridge Sequential Total Energy Package (CASTEP) based on the pseudopotential plane wave (PPW) approach was used to complete all density-functional theory (DFT) computations. The ultrasoft potentials (USP) were

used to characterize electron-ion interactions. To extend the wave functions with a cutoff kinetic energy of 480 eV, a plane-wave basis set was used. The functional parametrized by Perdew-Burke-Ernzerhof (PBE), a variant of the general gradient approximation (GGA), was utilized throughout for electron-electron exchange and correlation interactions.

All atom positions were permitted to relax throughout the geometry optimizations. Brillouin-zone integrations were performed in this study utilizing Monkhorst-Pack (MP) grids of special points separated by 0.08 $Å^{-1}$. The electronic self-consistent field (SCF) loop's convergence criteria was set to 2×10^{-6} eV/atom. The remaining forces in the atomic structures were reduced to less than 0.05 $eVÅ^{-1}$. All adsorption energy(ΔE) used in this paper were defined as：

$$\Delta E = E_{total} - E_{Mn3O4(211)} - E_{adsorbate}$$

where the E_{total} is the total energy of the system, $E_{Mn3O4(211)}$, $E_{adsorbate}$ are the energies of Mn_3O_4 (211) surface and isolated adsorbate, respectively. O_2^- and O_2 are almost the same in structure, but different in energy.

Electron paramagnetic resonance (EPR) analysis

5,5-dimethyl-1-pyrroline-N-oxide (DMPO, Sigma, USA) was used to trap free radicals (•OH and O_2^-) with short half-lives. The •OH scavenging ability of nanozyme was determined by incubating H_2O_2 (2.5 mM) in the presence of Fe^{2+} (2.5mM), followed by the addition of nanozyme (final concentration; 0, 5, 10, 20 μg/mL) and DMPO (200 mM). O_2^- was generated by the oxidation of xanthine (X) using xanthine oxidase (XO) [5]. The sample contained a final concentration of X (0.5 mM), XO (0.4 mg/mL), nanozyme (0, 5, 10, 20 μg/mL), and DMPO (100 mM) in PBS buffer. All the reactions were carried out in 50 mM phosphate buffer at pH 7.4 and the EPR signal was recorded after 5 min of incubation.

Superoxide dismutase (SOD) assay

The Fridovich assay was used to probe the SOD activity of nanozyme and natural SOD[5]. The SOD enzyme catalyzes O_2^- dismutation. The XO process mediated by xanthine oxidase, creates O_2^- which react with nitroblue tetrazolium (NBT). The solution changes color from yellow to blue when it reacts with the radicals. In the presence of SOD or nanozyme, the generated O_2^- are scavenged, reducing the amount of blue product as well as the intensity of blue color, which can be monitored by microplate reader.

In a typical measurement, 20 uL X (25 mM) and 20uL NBT (15 mM),10 μL nanozyme (1mg/mL) were added to PBS, followed by addition of a portion of PBS (0.1 M, pH 7.4) to obtain 900 uL sample. The centrifuge tube was then vortexed for 5 seconds. Finally, 100 uL of XO (1.5 mg/mL) was added to the sample before vorting for 5 seconds. The absorbance with time was recorded for 5 min at 565 nm wavelength. The inhibition curve is constructed by plotting the inhibition of the radical-NBT reaction for each sample against the final nanozyme concentration in the corresponding sample. The inhibition can be calculated using the formula below.

$$\text{SOD activity (\%)=Inhibitation (\%)} = \frac{\Delta A_0 - \Delta A_s}{\Delta A_0} \times 100\%$$

Where ΔA_S is the change in absorbance during the 5 min measurement time and ΔA_0 is the averaged value of the absorbance change for the blank samples (no nanozyme and natural enzyme added). During the assays, light scattering by the particles does contribute to the absolute value of absorbance, however, this factor was eliminated by taking the relative increase in the absorbance in the individual experiments.

Catalase assay; The CAT-like activity of nanozyme and natural CAT was measured using the generated O_2 using a specific oxygen electrode on Seven2Go Pro Analyzer (Mettler Toledo, China). The kinetic assays of nanozyme (0 - 4 μg/mL) and CAT (positive control,0 - 40 μg/mL) with H_2O_2 as the substrate were performed in 1.0 mL buffer solution (0.1 M PBS, pH 7.4). The generated O_2 was measured at reaction of 30s.

Glutathione peroxidase GPx; The GPx-like activity of nanozyme (10 μg/mL) and GPx (positive control, 10 μg/mL) was investigated using the classical Glutathione reductase (GR)-coupled assay by spectrophotometrically following the decrease in the concentration of nicotinamide adenine dinucleotide phosphate (NADPH) at 340 nm (the molar extinction coefficient of NADPH: ε340= 6.22 mM^{-1} cm^{-1}) on a microplate reader operating under kinetic mode[6]. In a typical experimental assay (total capacity ;1 mL), nanozyme (10 μg/mL), reduced glutathione (GSH) (5 mM), NADPH (0.2 mM), GR (3 units), H_2O_2 (0-200 mM) in PBS and the reaction was followed for 5 min at 37 °C water bath. The control reactions were performed in the absence of at least one of the reactants. The steady-state kinetics was studied by varying the concentration of H_2O_2.

The kinetics of all enzymatic reactions were calculated using the three standard Michaelis-Menten equations.

$$A = kbc \quad \text{(a)}$$

$$v_0 = \frac{V_{\max} \cdot [S]}{K_m + [S]} \quad \text{(b)}$$

$$\frac{1}{v_0} = \frac{K_m}{V_{\max}} \cdot \frac{1}{[S]} + \frac{1}{V_{\max}} \quad \text{(c)}$$

Note; Due to the lack of standardization in catalytic activities of nanozymes analysis, we provided relevant drug detailed information and procedured as much as possible to facilitate subsequent researchers to repeat related experiments.

Xanthine (X7375), Xanthine oxidase (X1875, ≥0.4 units/mg), Glutathione (PHR1359), Glutathione reductase (G3664), NADPH (10107824001), Nitroblue tetrazolium (N6876), Catalase (C9322, 2000-5000 units/mg), Superoxide dismutase (S9697, ≥2500 units/mg), Glutathione peroxidase (G6137, ≥300 units/mg) were obtained from Sigma. H_2O_2 was purchased from Shanghai Lingfeng Chemical Reagents Co., Ltd. (China).

Cells and animals

HL-60 line was purchased from the Shanghai Cell Bank of the Chinese Academy of Sciences (Shanghai, China). Neutrophils with chemotactic properties were considered differentiated successfully after five days of culture in 1.5% DMSO (Sigma, USA).

HUVEC cell line was obtained as a generous gift from Dr. Guangli Suo from Suzhou Institute of Nano-Tech and Nano-Bionics, Chinese Academy of Sciences

Neuron stem cells (NSCs) were isolated from postnatal rat telencephalons within 24 h of birth and were cultured as described previously[7].

Bone marrow-derived macrophages (BMDMs) were extracted from 80-100g female Sprague Dawley (SD) rats rat according to previously reported protocol[8]. Briefly, cells taken from rat femurs and tibiae were cultured in 5ng/ml G-CSF for one day, followed by 30ng/mL for next six days, which were considered M0-phase macrophages. LPS and IFN-r were utilized to stimulate M1 macrophage phenotype. IL-4 was used to stimulate M2 macrophage phenotype.

SD rats (160-180g) were purchased from Shanghai SLAC Laboratory Animal Co., Ltd. All animal experiments conformed to the National Institutes of Health guidelines and protocols were approved by the committee of Suzhou Institute of Nano-Tech and Nano-Bionics, the Chinese Academy of Sciences (Approval number: SINANO/EC/2022–070).

Cell viability

Cell viability was measured by the CCK-8 kit (Thermo Fisher Scientific, USA).

$$\text{Cell viability (\%)} = \frac{A_s\text{-}A_b}{A_c\text{-}A_b} \times 100\%$$

As: Absorbance of experimental wells (containing cells, medium, CCK-8 solution and drug solution). Ac: Absorbance of control wells (containing cells, culture medium, CCK-8 solution, without drug). Ab: Absorbance of blank wells (Containing medium, CCK-8 solution, without cells and drugs).

Target ability analysis

Migration chemotaxis of M@pMn assay was performed by applying 24-well Boyden chambers with 8 μm pore size polycarbonate membranes (Corning, USA) as described previously. HUVECs were seeded onto the upper chamber at $1x10^5$ cells, while the bottom chamber cultured with BMDMs. Different nanoparticles (pMn, $^{IRF-5}$*SiRNA*/pMn, M@pMn, $^{IRF-5}$*SiRNA*/M@pMn) were added to the upper chamber. After 24 h, the cells that remained on the upper side of the filters were removed using a cotton swab. The BMDMs of the lower chamber was collected for analysis of flow cytometry (FACS) and confocal laser scanning microscope (CLSM). Meanwhile, the obtained BMDMs also followed by treatment with 2.5% glutaraldehyde, and then the samples were gradiently eluted with a graded series of alcohol. The alcohol in the samples was then replaced with anhydrous propylene oxide or acetone, the epoxy resin was then infiltrated, and the catalyst was added to the epoxy resin in a ratio of 1.5% to 2% and mixed. After that, the samples were polymerized at 35°C for 12 h, 45°C for 12 h, 60°C for 24 h and ultrathin sectioned, finally observed with a transmission electron microscope (HT7700, Tokyo, Japan).

Intracellular oxidative stress assay

The BMDMs were seeded into the 24-well plates at a density of 1×10^5 cells per well, and allowed to adhere overnight. After treated nanoparticles for 6 h, the culture media were replaced by 1 mL DCFH-DA (10 μM/mL) and incubated at 37℃ for 20 min in the dark. The treated cells were washed three times with PBS (pH 7.4) and observed using CLSM.

Mitochondrial Depolarization Assay

Following the same treatment, BMDMs were then harvested and labeled with 10 μg/mL Rhodamine 123 at 37℃ for 10 min before being measured for fluorescence intensity using a FACS Calibur flow cytometer (Coulter Beckman, USA). SSC/FSC gating was used to filter debris, and 30,000 cellular events were captured.

Investigation of the polarization types of macrophages

The BMDMs were gently scraped down on ice with a cell scraper, washed three times with pre-cooled DPBS, and blocked for 45 min in DPBS containing 1% FBS and FcRblock (Biolegend, USA). Then the cells were stained CD11b and CD86 for 30min at 4℃. The cells were fixed and permeabilized with BD Fixation/Permeabilization Kit (BD Bioscience, USA) and, and then stained with CD206 for 30 min. The cells were followed by fluorescence intensity measurement using a FACS Calibur flow cytometer (Coulter Beckman, USA). Debris was filtered by SSC/CD11b gating with 30,000 cellular events collected. A description of related antibodies may be found in Supplementary Table 6.

Western blotting

The cells were lysed in RIPA buffer with a protease inhibitor cocktail (Yeasen, China) for 20 minutes on ice, centrifuged for 5 minutes at 1200 rpm to collect supernatant, and then boiled for 5 minutes in a metal bath at 100 °C. If there were animal tissue samples, then lysed the tissue homogenate for another 2h. Next, the total protein extracts (20 μg) were separated employing sodium dodecyl sulfate-polyacrylamide gel electrophoresis (SDS-PAGE) and then transferred onto a polyvinylidene difluoride (PVDF) membrane blocked with 5% (w/v) nonfat milk for 2 h. The PVDF membrane was washed with Tris-buffered saline with 0.1% Tween 20 detergent (TBST) buffer and then incubated overnight with primary antibodies and appropriate horseradish peroxidase (HRP)-conjugated secondary antibodies for 2 h. Finally, immunoreactive bands were developed using SuperSignal west femto maximum Sensitivity Substrate (Thermo Fisher Scientific, USA), and the relative protein quantity was normalized to the level of glyceraldehyde 3-phosphate dehydrogenase (GADPH).

Quantitative real-time polymerase chain reaction (RT-qPCR)

Total RNA was extracted with Trizol Reagent (Yeasen, China) per manufacturer’s recommendations. A total of 0.5 μg isolated RNA was reverse transcribed into cDNA with the cDNA synthesis kit (Yeasen, China). qPCR was performed according to the procedure specified by the reagent manufacturer (Yeasen, China). Samples were

normalized to Glyceraldehyde 3-phosphate dehydrogenase (Gapdh) and analyzed according to the delta/delta Ct (2^)-ΔΔCt)) method. All primer sequences are shown in Supplementary Table 5.

Gelatin hydrogels systhesis and characterization

Photocrosslinkable gelatin and photoinitiator were synthesized as described previously[9]. To prepare the gelatin hydrogels, nanozyme (10 μL, 10mg/mL) was mixed with a polymer solution of 10% w/v crosslinkable gelatin and 0.15% w/v photoinitiator (Lithium Phenyl (2,4,6-Trimethylbenzoyl) phosphinate, LAP). Then hydrogels quickly formed by UV curing lamp irradiation (Engineering For Life, China) for 40s.

The structures and morphologies of the samples were characterized. The morphology and microstructure of the hydrogels were determined using a field-emission SEM (Hitachi Regulus 8100, Japan). A rheometer (Kinexus pro$^+$ Instrument, Germany) was used to study the rheological properties of the prepared hydrogels with an 80-mm parallel plate configuration. A universal material testing machine (Instron, 3400, USA) was used to measure the hydrogels' compressive properties. The FTIR spectra of the hydrogels was recorded using Fourier transform infrared spectroscopy (FTIR, Thermo Scientific Nicolet iS5, USA).

Animal models

The rats were anesthetized with 10% chloral hydrate (0.5 g/kg). To expose the T9-T10 vertebrae, a vertical incision was made through the back muscle on each side of the vertebrae. Following a dorsal laminectomy, a 4±0.5 mm long T9 full lesion cavity was produced. When the bleeding stopped, the hydrogel was injected into the spinal cord cavity and cured in situ using a UV curing laser for 40 seconds. The back muscles and skin were stitched with degradable sutures. Manual bladder massage was done twice a day until the bladder resumed automatic urination. For 7 days and 8 weeks, eight animals were chosen at random to be sacrificed.

Immunofluorescence (IF) and immunohistochemistry (IHC)

Frozen sections of injured spinal cords were used for immunofluorescence staining and immunohistochemistry. The relevant primary antibodies were shown in Supplementary Table 6, and the secondary antibodies were all used by Invitrogen (USA).

Locomotor Function Test.

The open field (120×200 cm) Basso-Beattie-Bresnahan locomotor test was used to measure hindlimb motor function and was repeated weekly for 8 weeks after surgery. Individual rats were observed for at least 5 min by two independent observers who were blinded to the examined groups.

Software

Images were adjusted and quantified using the predetermined threshold setting in the Image J software. The particle size statistics of nanoparticles were carried out using Nano Measure software. Structure and visualization of related proteins are presented by PyMol Stereo 3D Zalman (pymol). The Michaelis-Menten saturation curve of catalytic abilities was fitted by origin. The raw data from the flow cytometer was further analyzed and plotted by FlowJo. Crystal visualization of DFT theoretical calculation was processed by VESTA.

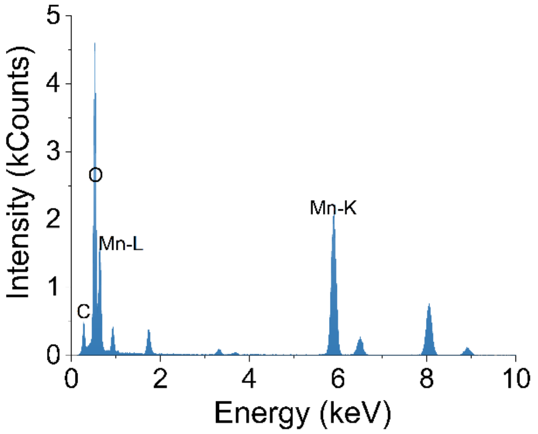

Figure S1. EDS spectrum of Mn_3O_4.

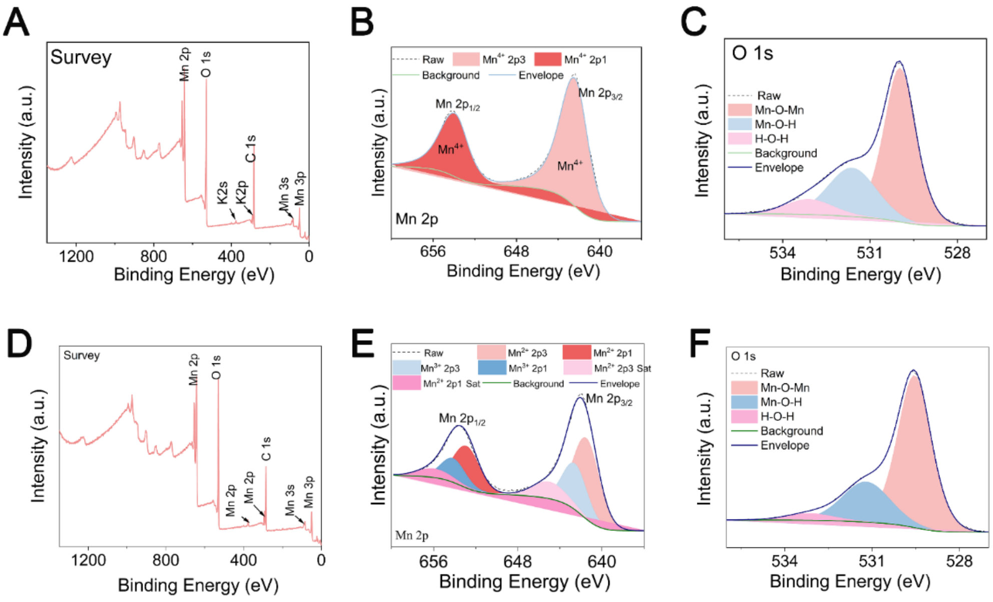

Figure S2. MnO_2 A) XPS survey spectrum of MnO_2. B) High-resolution Mn 2p spectrum, the peaks of Mn^{4+} $2p_{3/2}$ and Mn^{4+} $2p_{1/2}$ are fitted to energy components centered at around 642.4 and 653.9 eV. The atomic percentage of Mn^{4+} is 100%. C) High-resolution O 1s spectrum, the peaks of Mn-O-Mn and Mn-O-H are fitted to energy components centered at around 529.9 and 531.62 eV.

Mn_3O_4 D) XPS survey spectrum of Mn_3O_4. E) High-resolution Mn 2p spectrum, the peaks of Mn^{2+} $2p_{3/2}$, Mn^{2+} $2p_{1/2}$, Mn^{3+} $2p_{3/2}$ and Mn^{3+} $2p_{3/2}$ are 641.5, 652.9, 642.6,

and 654.2 eV. The atomic percentages of Mn^{2+} and Mn^{3+} are 65.32% and 34.68%. F) High-resolution O 1s spectrum, the peaks of Mn-O-Mn and Mn-O-H are fitted to energy components centered at around 529.6 and 531.2 eV.

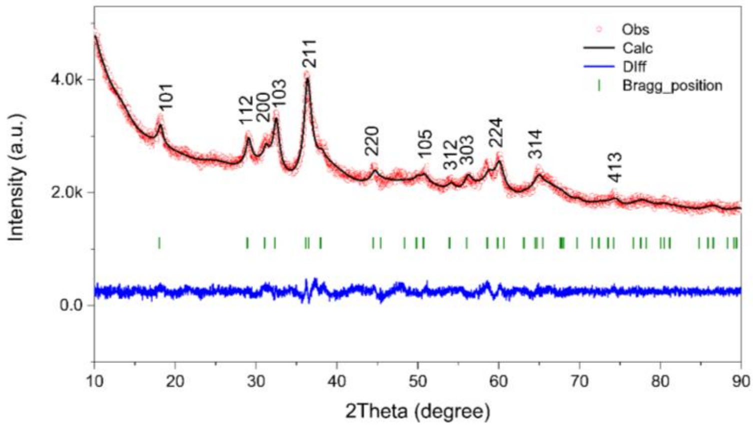

Figure S3. XRD patterns of Mn_3O_4, the crystal structure was optimized according to the 211 crystal plane of Mn_3O_4 for subsequent density functional theory (DFT) analysis.

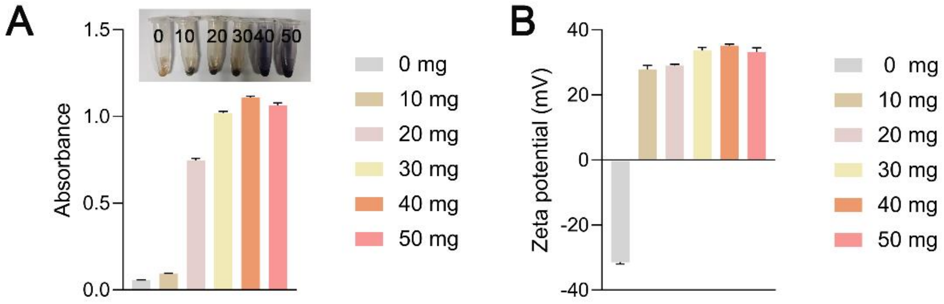

Figure S4. A) Absorbance of PEI coated on the surface of Mn_3O_4. The ninhydrin color reaction was used to determine the amount of PEI coupled with nanoparticles, as PEI had a number of amino groups that react with ninhydrin to produce a substance with the maximum absorption wavelength at 570 nm. B) Zeta potential of nanoparticles which Mn_3O_4 reacted with the different mass of PEI (0,10,20,30,40,50 mg).

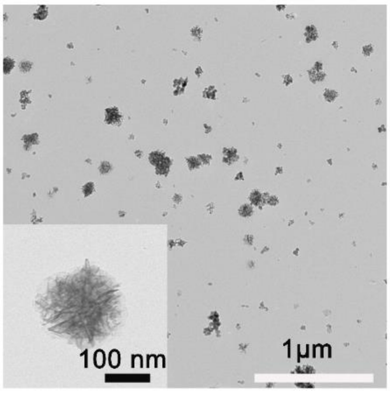

Figure S5. TEM image of PEI-Mn_3O_4. PEI coated havn't changed the nanoflower-like structure, indicating that the consistently high specific surface area was still suitable for gene delivery.

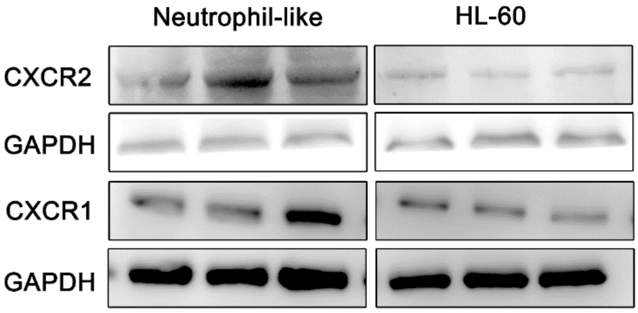

Figure S6. Western Blot of CXCR1 and CXCR2 in Neutrophil-like (treated with DMSO) and HL-60 cells. Three lanes in this western blot image reflect triplicate tests. The expression of CXCR1/2 in neutrophil-like cells was slightly higher than that in HL-60 cells, which was consistent with the results of TMT proteomic analysis.

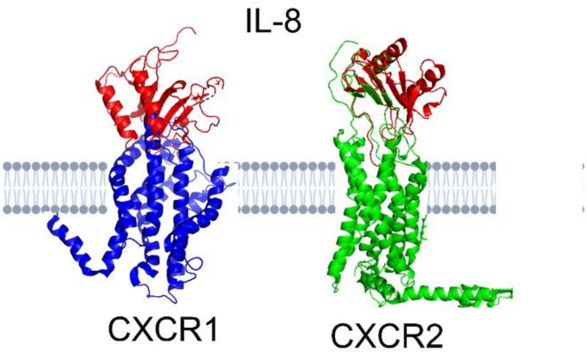

Figure S7. Computer simulation image of docking of CXCR1/2 to IL-8. Correlation sequences of CXCR1/CXCR2/IL-8 can be found in Supplementary Table 2 and 3.

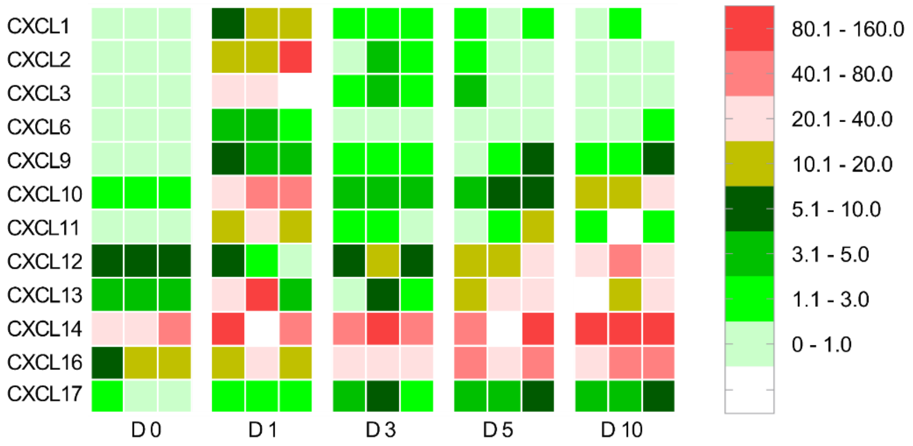

Figure S8. Heatmap of chemokine family (CXCL-family) gene expression in spinal cord tissue at diverse periods (0, 1, 3, 5, and 10 day) after SCI. The chemokine family remains highly expressed within 10 days of spinal cord injury. The relevant raw data can be found in Additional Sheet 2.

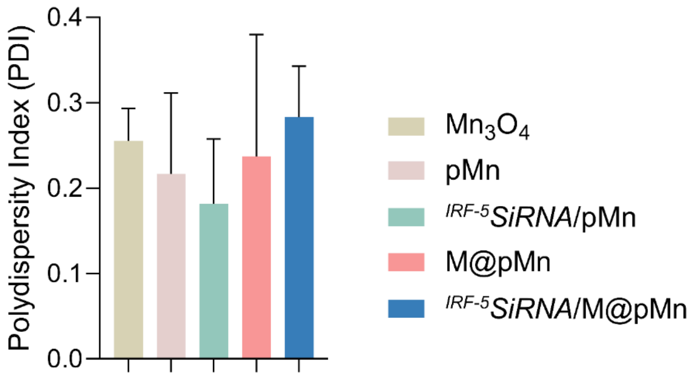

Figure S9. Particle size distribution (PDI) of different synthetic nanoparticles.

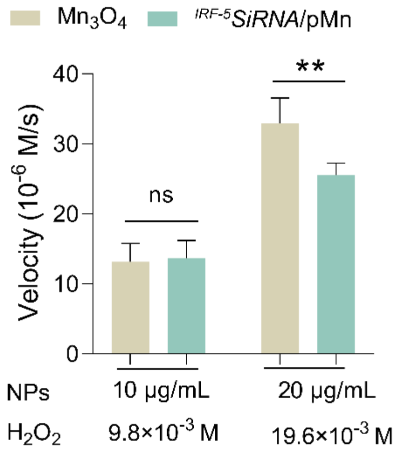

Figure S10. The plot indicates the ability of the nanoparticle to functionally mimic the CAT-enzyme with different concentrations of nanoparticles (Mn_3O_4 and ${}^{IRF\text{-}5}$*SiRNA*/pMn) and H_2O_2. The reaction mixture (1 mL) contained a fixed concentration of H_2O_2 and nanozyme in phosphate buffer (50 mM, pH 7.0). Under low concentration (10 μg/mL) conditions, Mn_3O_4 and ${}^{IRF\text{-}5}$*SiRNA*/pMn showed no significant difference in catalytic ability. However, under the concentration of 20 μg/mL, ${}^{IRF\text{-}5}$*SiRNA*/pMn showed lower catalytic ability than Mn_3O_4 due to the loading of siRNA.This phenomenon can be explained that at low concentrations, the difference in catalytic efficiency of the nanozymes was too small to be captured. At high concentrations, the difference in in catalytic efficiency was much larger, which can be detected significantly.

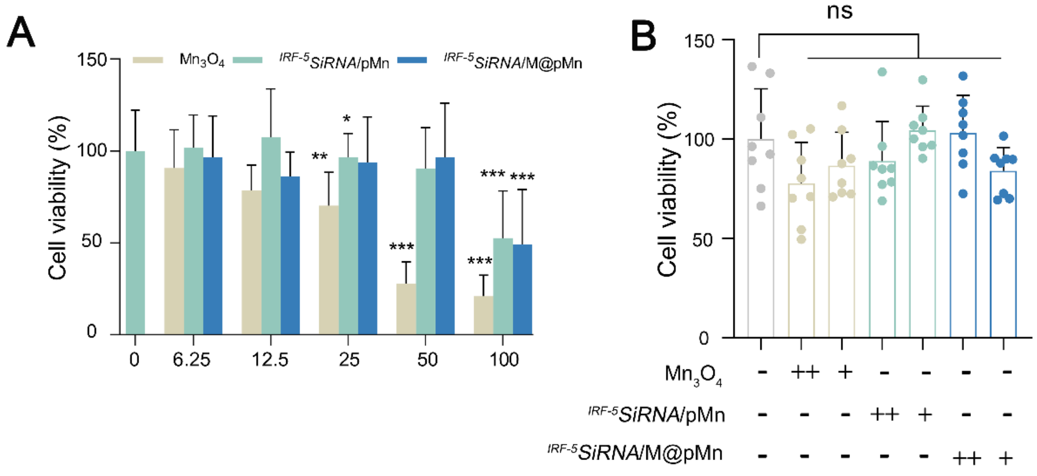

Figure S11. Cell viability of A) Bone Marrow-Derived Macrophages (BMDM) and B)

Neural Stem Cell (NSC) treated with nanoparticles (++ 25 µg/mL, +12.5 µg/mL).

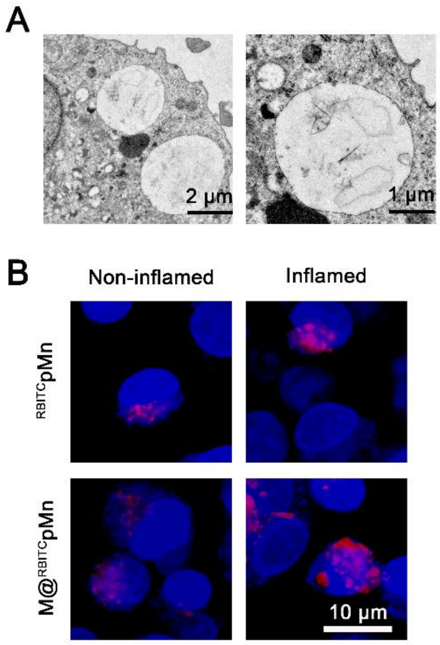

Figure S12. A) Bio-TEM images of BMDM cultured with pMn (concentration: 12.5 µg/mL). B) Representative confocal images of microglia (BV2 cells) incubated with RBITCpMn and M@ RBITCpMn with or without stimulation using LPS and IFN-γ (Blue, nuclei; red, RBITCpMn).

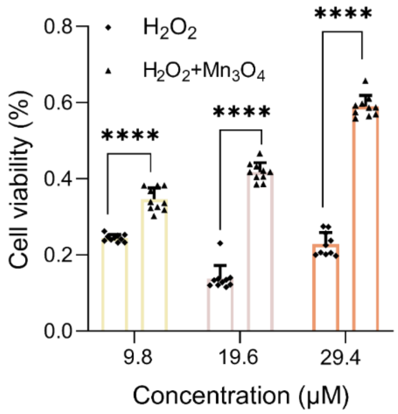

Figure S13. Cell viability of different concentrations of H_2O_2 treated with BMDM (M@pMn; 12.5ug/mL).

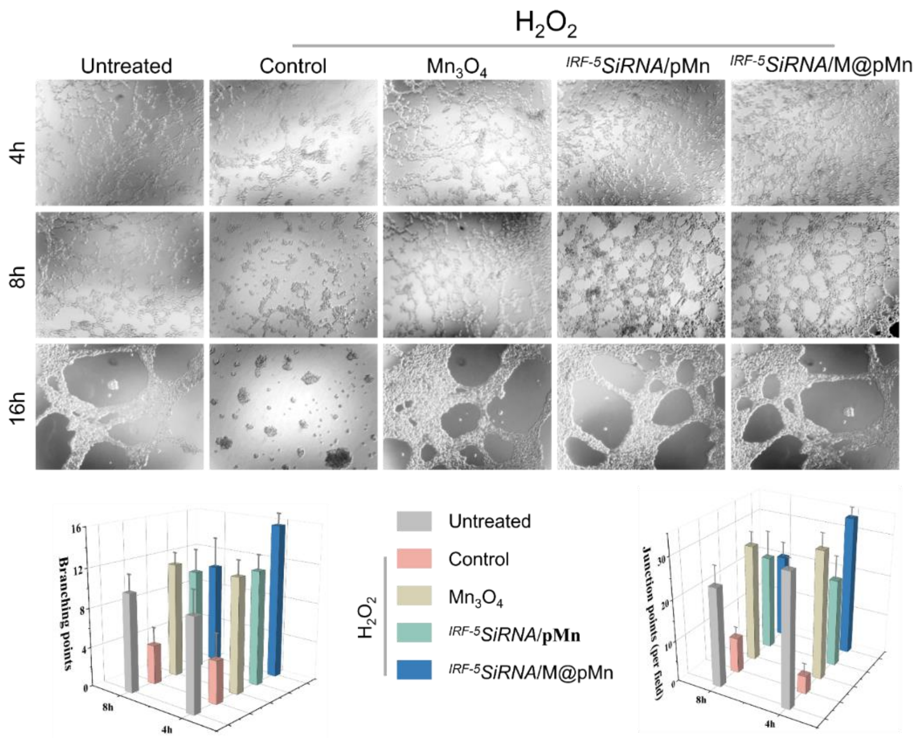

Figure S14. Angiogenesis assay of HUVEC in vitro, all images viewed under 10X objective. Branching points and Junction points were measured by the angiogenic analysis plugin for Image J.

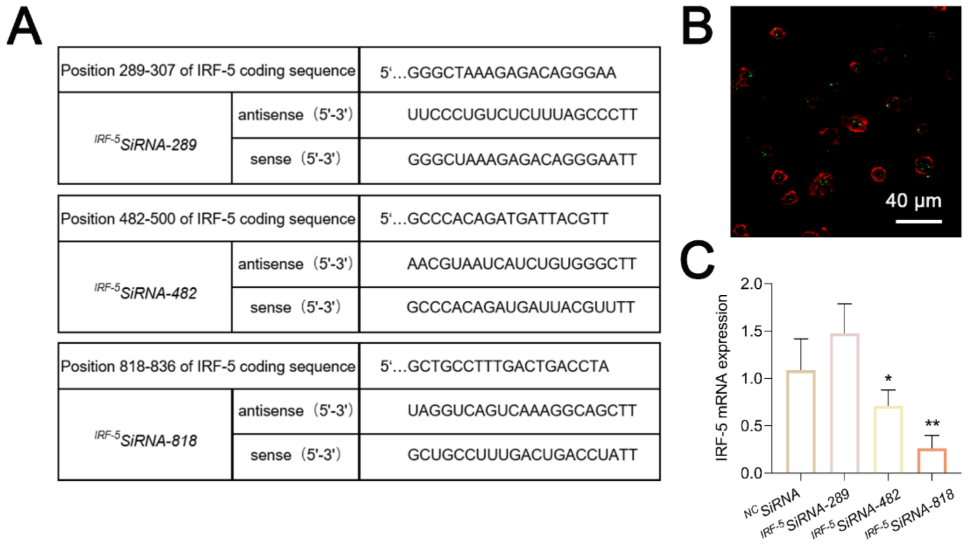

Figure S15. A) Designed SiRNA sequences (289, 482, and 818) that may have silencing effects. B) CLSM images showed SiRNA was successfully transfected into BMDM (Red; Dil of BMDM membrane, green; FAM labeled SiRNA). C) Silencing

efficiency was determined by measuring the decrease IRF-5 gene expression by qPCR.

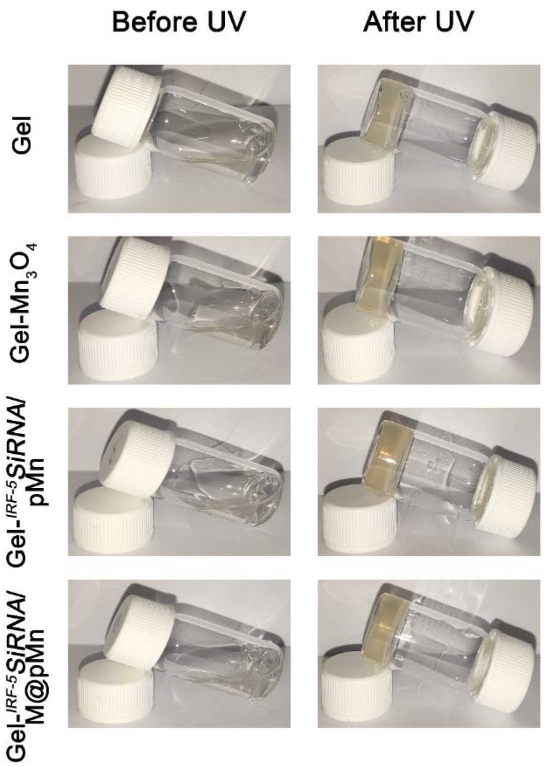

Figure S16. The picture of different gelatin hydrogels (before and after UV).

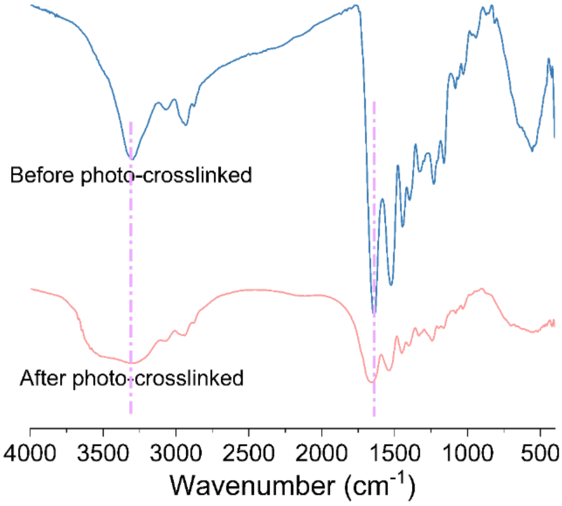

Figure S17. FTIR (Fourier Transform infrared spectroscopy) of functional hydrogel (before and after photo-crosslinked).

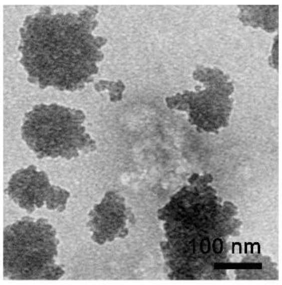

Figure S18. TEM image of M@pMn after being blended in gelatin hydrogel. The M@pMn was obtained by lysing gelatin hydrogel and collecting the supernatant.

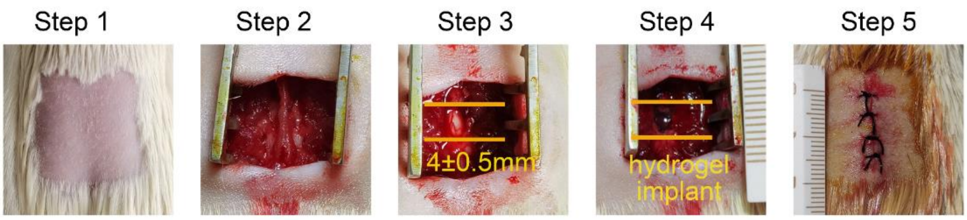

Figure S19. Pictures of spinal cord injury models in rats.

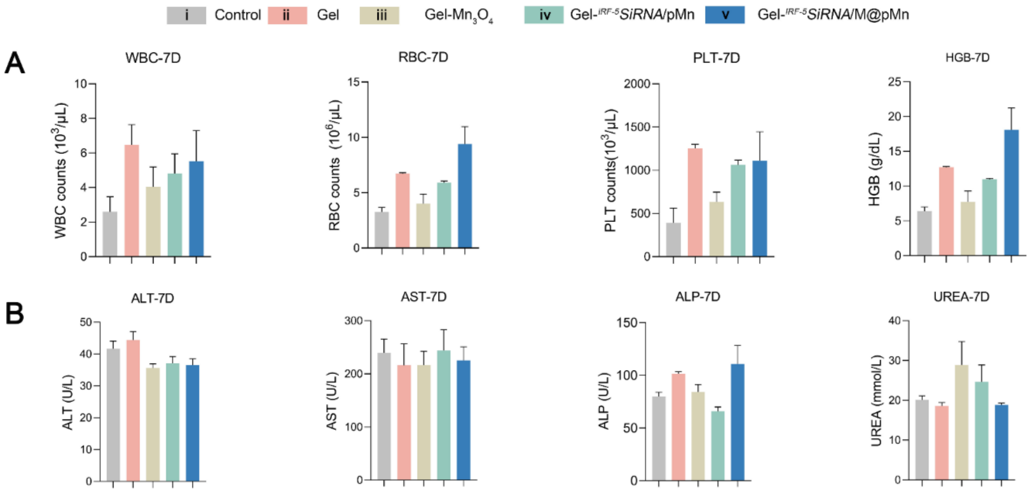

Figure S20. Blood was collected 7 days after treated rats were sacrificed. A) Blood cell analysis of WBC (white blood cells), RBC (red blood cells), PLT (platelets), and HGB (hemoglobin). B) Biochemical molecules of ALT (alanine aminotransferase), ALP (alkaline phosphatase), AST (aspartate aminotransferase), and UREA (creatinine).

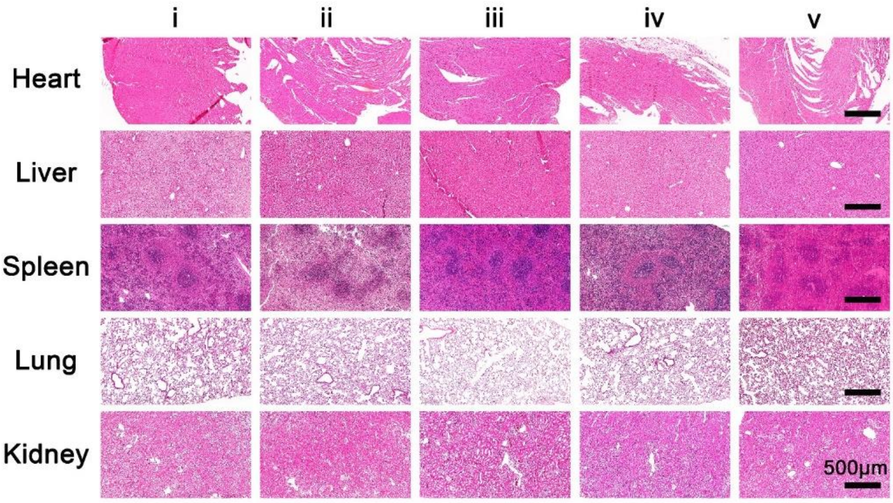

Figure S21. Representative images of H&E staining of main organs of SCI rats treated for 7 days. The rats were treated for 7 days with different hydrogels. (i; Control, ii; Gel, iii; Gel-Mn_3O_4, iv; Gel-$^{IRF\text{-}5}$*SiRNA*/ pMn, v; Gel-$^{IRF\text{-}5}$*SiRNA*/M@pMn).

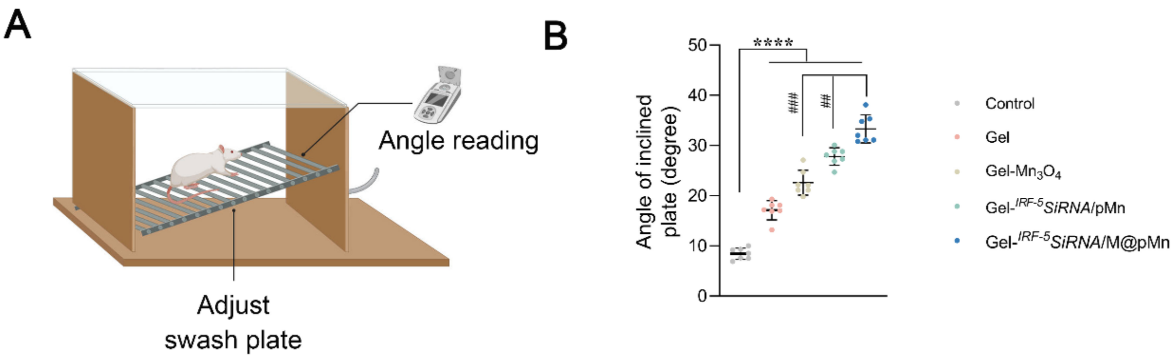

Figure S22. A) The treated rats were placed on a adjust swash plate to measure the recovery of the rat's hindlimb grasping function. The angle reading was measured by a digital inclinometer. B) The maximum angle at which the rats wouldn’t fall off.

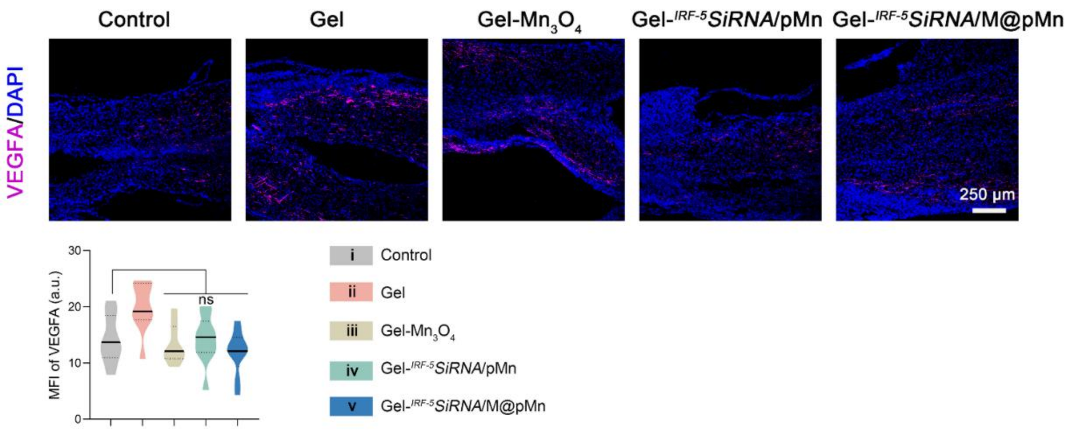

Figure S23. A) Representative immunofluorescence staining images of VEGFA and quantification analysis B) at the lesion site of SCI rat.

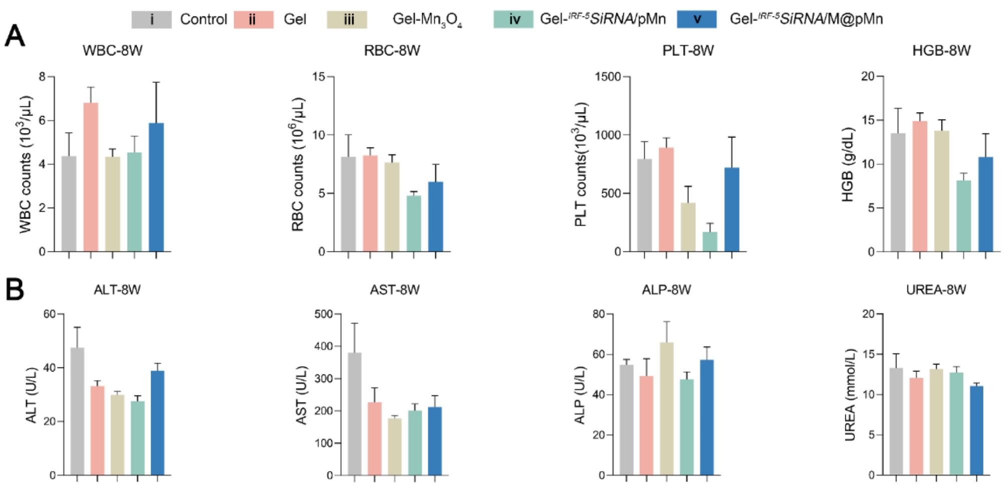

Figure S24. Blood was collected 8 weeks after treated rats were sacrificed. A) Blood cell analysis of WBC (white blood cells), RBC (red blood cells), PLT (platelets), and HGB (hemoglobin). B) Biochemical molecules of ALT (alanine aminotransferase), ALP (alkaline phosphatase), AST (aspartate aminotransferase), and UREA (creatinine).

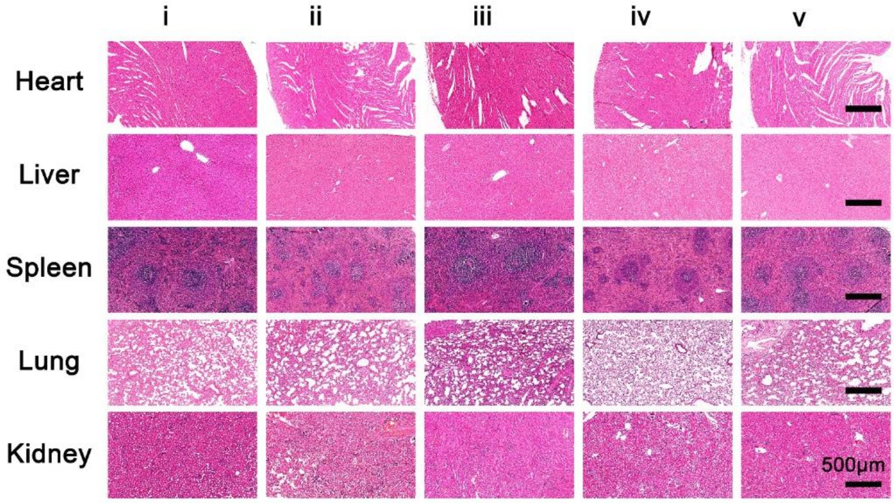

Figure S25. Representative images of H&E staining of main organs of SCI rats treated for 8 weeks. The rats were treated for 8 weeks with different hydrogels. (i; Control, ii; Gel, iii; Gel-Mn_3O_4, iv; Gel-$^{IRF\text{-}5}$*SiRNA*/ pMn, v; Gel-$^{IRF\text{-}5}$*SiRNA*/M@pMn).

Supplementary Table 1. CXCL1 CXCL2 CXCL3 (rat) protein amino acid sequence;

1) CXCL1;

NCBI Reference Sequence: NP_110472.2

mvsatrsllc aallllatsr qatgapvane lrcqclqtva gihfkniqsl kvmppgphct qteviatlkn

greacldpea pmvqkivqkm lkgvpk

2) CXCL2;

NCBI Reference Sequence: NP_446099.1

mapptrqlln avlvllllla tnhqgtgvvv aselrcqclt tlprvdfkni qsltvtppgp hcaqteviat

lkdghevcln peaplvqriv qkilnkgkan

3) CXCL3;

NCBI Reference Sequence: NP_001381519.1

mapptrrlln aalllllllm atshqpsgtv varelrcqcl ktlprvdfen iqsltvtppg phctqtevia

tlkdgqevcl npqaprlqki iqkllksdks s

Supplementary Table 2. IL-8 (human) sequence

GenBank: AAH13615.1

mtsklavall aaflisaalc egavlprsak elrcqcikty skpfhpkfik elrviesgph canteiivkl sdgrelcldp kenwvqrvve kflkraens

Supplementary Table 3. CXCR1 CXCR2 (human) sequence.

1) CXCR1

NCBI Reference Sequence: NP_000625.1

msnitdpqmw dfddlnftgm ppadedyspc mletetlnky vviiayalvf llsllgnslv mlvilysrvg rsvtdvylln laladllfal tlpiwaaskv ngwifgtflc kvvsllkevn fysgilllac isvdrylaiv hatrtltqkr hlvkfvclgc wglsmnlslp fflfrqayhp nnsspvcyev lgndtakwrm vlrilphtfg fivplfvmlf cygftlrtlf kahmgqkhra mrvifavvli fllcwlpynl vlladtlmrt qviqescerr nnigraldat eilgflhscl npiiyafigq nfrhgflkil amhglvskef larhrvtsyt sssvnvssnl

2) CXCR2

NCBI Reference Sequence: NP_001161770.1

medfnmesds fedfwkgedl snysysstlp pflldaapce pesleinkyf vviiyalvfl lsllgnslvm lvilysrvgr svtdvyllnl aladllfalt lpiwaaskvn gwifgtflck vvsllkevnf ysgilllaci svdrylaivh atrtltqkry lvkficlsiw glslllalpv llfrrtvyss nvspacyedm gnntanwrml lrilpqsfgf ivpllimlfc ygftlrtlfk ahmgqkhram rvifavvlif llcwlpynlv lladtlmrtq viqetcerrn hidraldate ilgilhscln pliyafigqk frhgllkila ihgliskdsl pkdsrpsfvg sssghtsttl

Supplementary Table 4. The kinetic parameter values obtained from Lineweaver-Burk plots of CAT-like activity and GPx-like activity of nanozyme.

		Substrate	Km	Vmax
CAT	Natural CAT	H_2O_2	0.01158±0.00174 M	17.89±0.2076 M/s
	Nanozyme	H_2O_2	0.04813±0.00249 M	17.09±0.2282 M/s
GPx	Natural GPx	H_2O_2	0.02292±0.0133 M	5.144±1.191 10^{-6} M/s
	Nanozyme	H_2O_2	0.07136±0.0277 M	4.701±0.9238 10^{-6} M/s

Supplementary Table 5. IRF-5 coding sequence.

Rattus norvegicus interferon regulatory factor 5 (Irf5), mRNA

NCBI Reference Sequence: NM_001106586.1

ATGAACCAGTCAGCCCCAGGGATTCCCACACCACCCCGCCGTGTGAGGCT
GAAGCCCTGGCTGGTGGCCC
AGGTGAACAGCTGCCAGTACCCAGGGCTCCAGTGGGTCAACGGGGAAAA
GAAACTCTTCTATATCCCCTG
GCGCCATGCTACAAGGCACGGTCCCAGCCAGGATGGAGACAACACCATCT
TCAAGGCCTGGGCTAAAGAG
ACAGGGAAGTACACGGAGGGCGTGGATGAGGCTGACCCAGCCAAATGGA
AGGCCAACCTGCGCTGTGCCC
TTAACAAAAGCCGTGACTTCCAGCTGTACTATGATGGCCCTCGAGACATG
CCACCTCAGCCCTACAAGAT
CTACGAGGTCTGCTCCAATGGCCCTGCTCCCACAGAGTCCCAGCCCACAG
ATGATTACGTTCTGGGAGAA
GAGGAGGAAGAGGAAGATGAAGAGCTCCAGAGAATGCTACCAGGCCTGA
GTATCACAGAACCTGTGCTAC
CTGGGCCTCCCATAGCACCCTATTCCTTACCCAAAGAAGACACCAAGTGG
CCACCTGCTCTCCAGCCACC
TGTAGGGCTGGGTCCCCCTCCCCCAGACCCAAATCTCCTGGCCCCTCCCTC
TGGAGATTCTGCTGGCTTT
AGGCAGCTTCTCCCTGAGGTCCTGGAGCCTGGACCCCTGGCTTCCAACCA
GCCCCCTACAGAACAACTCT
TGCCTGACCTGCTCATCAGCCCCCACATGCTGCCTTTGACTGACCTAGAGA
TCAAGTTCCAGTACCGGGG
ACGTCCACCGCGGGCCCTCACCATCAGCAACCCACAAGGCTGCAGGCTCT
TCTATAGCCAGCTAGAGGCT

ACCCAGGAGCAAGTGGAACTCTTTGGCCCTGTGACCCTGGAGCAAGTTCG

CTTCCCTAGCCCAGAGGATA

TCCCCAGTGACAAGCAGCGCTTCTATACGAACCAGCTGCTGGATGTCCTG

GACCGAGGGCTCATCCTGCA

GCTGCAGGGCCAGGACCTGTATGCCATCCGTCTGTGCCAGTGTAAGGTGT

TCTGGAGTGGGCCCTGTGCC

TTGGCCCATGGTTCCTGCCCCAACCCCATCCAGCGGGAAGTCAAGACAAA

GCTCTTTAGCCTAGAGCAGT

TTCTCAATGAACTCATCCTGTTCCAGAAGGGCCAAACCAATACCCCACCA

CCTTTTGAGATCTTCTTTTG

CTTTGGAGAAGAATGGCCTGACCTCAAACCCCGAGAGAAGAAGCTCATTA

CTGTACAGGTGGTACCTGTC

GCAGCCCGGTTGCTGCTGGAGATGTTCTCAGGAGAGCTTTCTTGGTCAGC

AGACAGCATTCGGCTGCAGA

TCTCAAACCCGGATCTCAAAGACCACATGGTAGAGCAGTTCAAAGAGCTC

CATCACATCTGGCAGTCCCA

GCAGCGCTTGCAGCCCATGGTCCAGGCCCCTCCTGTGGCAGGCCTTGATG

CTGGCCAGGGGCCCTGGCCC

ATGCACCCAGTTGGCATGCAATAG

Supplementary Table 5. Primers used for qPT-PCR.

**Gene name**	**Primer (Forward)**	**Primer (Reverse)**
IRF-5 (cell)	ATGCTGCCTCTGACCGA	GCCGAAGAGTTCCACCTG
IRF-5 (tissue)	AGATCTACGAGGTCTGCTCC	TCAGGCCTGGTAGCATTCTC
CD86	GACACCCACGGGATCAATTA	GCCTCCTCTATTTCAGGTTCAC
CD206	ACTGCGTGGTGATGAAAGG	TAACCCAGTGGTTGCTCACA
TNF-α	GACCCTCACACTCAGATCATCTTCT	TGCTACGACGTGGGCTACG
IL-1β	TCCACAGCCACAATGAGT	TCCACAGCCACAATGAGT
TGF-β	TGGCCAGATCCTGTCCAAAC	GTTGTACAAAGCGAGCACCG
IL-10	TTGAACCACCCGGCATCTAC	CCAAGGAGTTGCTCCCGTTA
CXCL1	AGACAGTGGCAGGGATTCAC	AAGCCTCGCGACCATTCTTG
CXCL2	TCCTCAATGCTGTACTGGTC	TGAAGTCAACCCTTGGTAGG
CXCL3	TCAATGCTGCACTGCTTCTG	TCCTTGAGAGTGGCTATGAC
CXCL4	TTCTTCTGGGTCTGCTGTTG	TGCGTTTGAGATGGATCCTG
CXCL6	TTTCTGCTGCTGTTCACACTG	TAGCTATGACTTCCACCTTGG
CXCL7	TTCAGACTCAGACCTACATC	AATGGCTCGTTGTTATCAGG
Tuj1	GAGGCCTCCTCTCACAAGTA	GTGAGTGAGTGAGCTGGAAG
Dcx	ACCAAGACGCAAATGGAACT	AGCCTTGTTGGTCACTTGAG
Stmn1	GACGGCAGGGTAGGGAG	CAACTTAGTCAGCCTCGGTC
Map2	CGTTCTCTCTGAACAGCTCC	TTCCTGGTGTCCTGGGATAG
Sox10	CTGCTATCCAGGCTCACTAC	GATGTCCACATTGCCGAAGT
Chat	AAGCAACAAGAAGCTCGTCA	GATGGATGCGCTCTCATAGG

Mnx1	GGCGCTTTCCTACTCGTATC	ACTTCCCCAAGAGGTTCGAT
Isl2	GACTACGTCAGGCTGTTCG	TCAAGCCGGTCATCTCTACC
Htr3a	CGTGGATGAGAAGAACCAGG	GCTGTAGGGGCTTGTAGTTC
Calbindin	AGATGGCCAGGTTACTACCA	GCCACCAACTCTAGTTGTCC
Gad2	GCATCGGAAACAAGCTATGC	TACAGGGGCGATCTCATAGG
α-SMA	CCTCTTCCAGCCATCTTTCAT	CGAGAGGACGTTGTTAGCATAG
VEGFA	GAAGACACAGTGGTGGAAGAAG	ACAAGGTCCTCCTGAGCTATAC
CD31	GCCGTCAAATACTGGGTTAGT	GCACTGTACACCTCCAAAGAT
Vcam1	AGCAGACAGCTAAAGAACGG	GGCACACTTCCACAAGTACA
Gapdh	GAAGCTCATTTCCTGGTATGACA	ATTGATGGTATTCGAGAGAAGGG

Supplementary Table 6. Primary antibodies.

Antibody	Host animals	Source and Reagent Cat. No.	Dilution	Application
CXCR1	Rb	Biorbyt, orb10487	1;200	WB
CXCR2	Rb	Abcam, ab65968	1;1000	WB
ATPase Na^+/ K^+ beta 2	Rb	Bioss, bs-1152R	1;200	WB
GAPDH	Ms	Abcam, ab8245	1;1000	WB
IRF-5	Rb	Abcam, ab181553	1;1000 (WB)<br>1;1000 (IF)	WB, IF
CD86-FITC	Rb	Biossusa, BS-1035-FITC	1;200	FACS
MRC-PE	Rb	Biossusa, BS-4727-PE	1;200	FACS

CD11b-PE/Cy7	Ms	Biolegend, 201817	1;500	FACS
NF-κB	Rb	CST, 8242s	1;1000	WB
pNF-κB	Rb	CST, 3033s	1;1000	WB
IκB	Rb	CST, 9242s	1;1000	WB
pIκB	Ms	CST, 9246s	1;1000	WB
Tuj1-488	Ms	Abcam, ab195879	1;500	IF
GFAP-647	Rb	Abcam, ab194325	1;500	IF
HIF-1α	Rb	Abcam, ab51608	1;100	IHC
CD86	Ms	Abcam, ab238468	1;200	IF
CD206	Rb	Abcam, ab64693	1;500	IF
F4/80	Rb	Abcam, ab300421	1;800	IHC
CD68	MS	Abcam, ab31630	1;800	IHC
ChAT	Ms	Sigma, AMAB91130	1;400	IF
Calbindin	Rb	Abcam, ab108404	1;150	IF
Brn3a	Rb	Abcam, ab245230	1;100	IF
Tuj1	Rb	Abcam, ab18207	1;1000	IF
Map2	Rb	Abcam, ab32454	1;1000	IF
GFAP	Chk	Abcam, ab4674	1;1000	IF
CD31	Ms	Proteintech, 66065-2	1;400	IF
VEGFA	Rb	Affinit, AF5131	1;200	IF

Rb; rabbit, Ms; mouse, Chk; chicken

WB; Western Blot, FACS; Fluorescence Activated Cell Sorter,

IF; (immunofluorescence), IHC; (immunohistochemistry)

Reference

[1] N. Singh, M. A. Savanur, S. Srivastava, P. D'Silva, G. Mugesh, *Angew Chem Int Ed Engl* **2017**, 56, 14267.

[2] M. Z. Zou, W. L. Liu, C. X. Li, D. W. Zheng, J. Y. Zeng, F. Gao, J. J. Ye, X. Z. Zhang, *Small* **2018**, 14, e1801120.

[3] a)J. Sarmiento, C. Shumate, K. Suetomi, A. Ravindran, L. Villegas, K. Rajarathnam, J. Navarro, *PLoS One* **2011**, 6, e27967; b)S. Kharche, M. Joshi, A. Chattopadhyay, D. Sengupta, *PLoS Comput Biol* **2021**, 17, e1008593.

[4] I. T. Desta, K. A. Porter, B. Xia, D. Kozakov, S. Vajda, *Structure* **2020**, 28, 1071.

[5] N. B. Alsharif, K. Bere, S. Saringer, G. F. Samu, D. Takacs, V. Hornok, I. Szilagyi, *J Mater Chem B* **2021**, 9, 4929.

[6] K. P. Bhabak, G. Mugesh, *Chemistry* **2007**, 13, 4594.

[7] X. Li, S. Liu, Y. Zhao, J. Li, W. Ding, S. Han, B. Chen, Z. Xiao, J. Dai, *Advanced Functional Materials* **2016**, 26, 5835.

[8] H. Tan, Y. Song, J. Chen, N. Zhang, Q. Wang, Q. Li, J. Gao, H. Yang, Z. Dong, X. Weng, Z. Wang, D. Sun, W. Yakufu, Z. Pang, Z. Huang, J. Ge, *Adv Sci (Weinh)* **2021**, 8, e2100787.

[9] H. Shen, B. Xu, C. Yang, W. Xue, Z. You, X. Wu, D. Ma, D. Shao, K. Leong, J. Dai, *Biomaterials* **2022**, 280, 121279.
